# Epidemiology and Risk Modelling of Influenza A Virus Within and Between Pig Herds in Northern Lao PDR

**DOI:** 10.1155/tbed/2407533

**Published:** 2026-01-23

**Authors:** Arata Hidano, William T. M. Leung, Souk Phomhacksa, Anna Durrance-Bagale, Jose A. Garcia-Rivera, Anca Selariu, Robert D. Hontz, Andrew G. Letizia, Watthana Theppangna, James W. Rudge

**Affiliations:** ^1^ Communicable Diseases Policy Research Group, London School of Hygiene and Tropical Medicine, London, UK, lshtm.ac.uk; ^2^ National Animal Health Laboratory, Department of Livestock and Fisheries, Ministry of Agriculture and Forestry, Vientiane, Laos; ^3^ U.S. Naval Medical Research Unit INDO PACIFIC (NAMRU INDO PACIFIC), Sembawang, Singapore

## Abstract

Animal‐origin influenza A virus (IAV) is a perennial candidate for causing the next pandemic. With high risk for interspecies IAV transmission but limited resources for surveillance, particularly in rural areas of low‐ and middle‐income countries (LMICs) such as Laos, there is a need to develop targeted, risk‐based strategies for early detection of novel IAVs that may emerge in pigs. We conducted (1) a cross‐sectional survey to characterise pig producer types, management practices and pig movement patterns; (2) sampling among pigs in slaughterhouses to quantify IAV seroprevalence and infection; and (3) within‐ and between‐herd disease modelling exploring the relative importance of farm type for the IAV epidemiology. Overall, 31.3% (100/319) of sera and 1.4% (7/515) of nasal swab samples from pigs tested positive for IAV antibodies (ELISA) and viral RNA (PCR detection of IAV M‐gene), respectively. Most pigs sampled were exotic breeds and supplied by commercial farms. Using hierarchical Bayesian logistic regression models, seropositivity was significantly higher among exotic breeds compared with local breeds and higher among pigs originating from provinces outside of our study area. Stochastic, individual‐based models of within‐ and between‐herd transmission were developed and calibrated for five pig producer types using the cross‐sectional data from 202 study participants. The modelling results suggested sustained IAV transmission between farms was unlikely unless the probability of local transmission, independent of pig movement, was relatively high, and the initial infection was seeded in areas with higher densities of smallholders. Between‐herd IAV transmission was only sustained in scenarios where persistently infected commercial farms were present to continuously seed infection among the pig smallholder network. Together, these factors underscore risks associated with livestock intensification in commercial and smallholder productions. A larger study is warranted to fully characterise the interprovincial pig movement and evaluate IAV transmission within Laos to inform the national surveillance strategy.

## 1. Introduction

Influenza A viruses (IAVs) are a perennial candidate for causing the next pandemic, with the emergence of new strains and subtypes facilitated by interspecies IAV transmission [1]. In Southeast Asia (SEA), the increasingly high densities of poultry, pig, and human populations living in close proximity to each other, combined with elevated levels of avian IAV (avIAV) circulation, create many opportunities for animal‐to‐human spillover to occur [2]. With limited resources for animal IAV surveillance, particularly in low‐ and middle‐income countries (LMICs), there is a need to develop risk‐based strategies that target subpopulations where the risk of novel IAV emergence and transmission is highest.

A wide range of IAV subtypes circulate in avian reservoirs, and while several of these, including highly pathogenic avian influenza (HPAI) A/H5N1 and, more recently, A/H7N9, cause sporadic and often fatal disease in humans, there is limited evidence of sustained transmission between humans [3]. Instead, efficient human‐to‐human transmission requires avIAVs to initially adapt successfully to mammals [4]. Pigs, having cell receptors which can bind both to avIAVs and mammalian IAVs [5], are considered important intermediate mammalian hosts which can facilitate such adaptation. With frequent bidirectional IAV transmission between pigs and humans [6, 7], novel IAVs adapted to pigs may readily transmit to humans, potentially causing an outbreak. The identification of a novel lineage of the Eurasian Avian–like H1N1 swine influenza virus (EA G4 H1N1) in China and concerns surrounding the pandemic potential of this virus [8] further underscores the urgency for increased surveillance and research on influenza in pigs. This is especially important in LMICs of the Lower Mekong region, such as Laos, where geographic proximity and trade with China heighten the risk of transboundary spread of novel IAVs [9]. Such vulnerabilities are further compounded by human and veterinary public health systems which are underresourced to respond to emerging outbreaks.

Designing effective, risk‐based surveillance strategies for early detection, prevention, and mitigation of novel IAVs requires in‐depth understanding of multiple risk ‘components’ and how these risks converge in time and space and along livestock value chains [10]. Within a swine farm, risk of reassortment between avian and swine IAVs (swIAVs) and adaptation of their progenitor viruses will depend on the frequency of IAV introductions and duration of IAV persistence at that site [6], both of which are facilitated by limited biosecurity practices in LMICs [2, 11, 12]. Persistence of swIAVs will also depend on factors such as the presence of maternal antibodies, introduction of susceptible pigs, and herd sizes and structures. Although evidence is limited, studies conducted in Vietnam suggest that subtypes of swIAV differ between animal smallholders and larger commercial farms [13, 14]. In addition to intraherd IAV dynamics, one must also consider the role of interherd transmission for persistence of IAV in a pig production system. Together, these highlight the importance of understanding characteristics of pig production systems as well as contact structures between farms that are likely to affect swIAV dynamics in low‐income settings, on which there is a severe lack of published research.

Laos is a landlocked country, which shares its border with China, Vietnam, Cambodia, Myanmar and Thailand. With this geographical significance, while epidemiological and genetic information on avIAV has been collected in Laos, such information on swIAV remains scarce. The most recent (and indeed only) previous epidemiological study we could identify on swIAV in Laos reported a seroprevalence of 1.8% among pigs sampled at slaughterhouses and killing points in 2008, indicating a low intensity of swIAV circulation at that time [15]; however, data on variables which may be associated with swIAV risks such as the origin of pigs were not collected, making it difficult to quantify the risk of swIAV infections in different pig populations. By combining a cross‐sectional study of pig producers, biological slaughterhouse sampling from pigs, and mathematical modelling approaches, we aimed to identify the typology of pig producers in northern Laos and characterise the nature of swIAV transmission within and between pig producers to inform the design of a risk‐based swIAV surveillance in this region.

## 2. Materials and Methods

### 2.1. Overall Study Design

Questionnaire surveys were used to capture demographic and management parameters (such as between‐farm pig movement frequencies) relevant to within‐ and between‐IAV transmission. Slaughterhouse sampling was conducted to quantify the IAV infection and exposure risk across different farm types. We initially intended to capture pig movement parameters from farms to slaughterhouses to inform the abovementioned mathematical model; however, this was infeasible as pigs were transported by traders who could not inform the pig origin. The result of the slaughterhouse sampling was therefore not used to inform the mathematical model and instead literature and range of a theoretical values were used to explore the impact of these parameters as explained below and in the supporting information.

### 2.2. Study Population

Two provinces in northern Laos, Oudomxay and Luang Namtha, were selected for this study based on the potential diversity of pig production systems in these regions, proximity to China, and a reported history of importation of pigs from neighbouring Thailand and Vietnam which present risks for transboundary spread of pathogens such as IAV. Furthermore, there exist gaps in knowledge on production systems and disease risks in such provinces (which have been relatively understudied), along with limited surveillance systems in remote areas located away from main urban centres. These provinces are therefore particularly important for livestock value chain and risk analyses to inform surveillance strategies.

Within our study provinces, Xay district (Oudomxay province) and Namtha district (Luang Namtha province) were initially selected for the cross‐sectional survey following a rapid situational analysis, involving qualitative interviews with key stakeholders, which indicated the presence of both commercial pig farms and smallholders (at least before the African Swine Fever [ASF] outbreak, although no commercial farms were identified to be operating in Luang Namtha during the subsequent field surveys). Additionally, some commercial farms and smallholders in Ben district (Oudomxay province) were recruited as they were located close to Xay district.

### 2.3. Questionnaire Survey

As the list of pig raisers in this region was not available, we took the following strategy to identify as diverse pig production types as possible. Provincial and district veterinarians were asked to list all commercial pig farms and any smallholders known to keep sows or boars in the area. We prioritised villages with these production types because our preceding qualitative study (data unpublished) suggested these types had more diverse farm practices and more frequent pig movements. We then contacted gatekeepers of these villages, such as village chiefs, to identify all pig raisers in the village. We visited the pig raisers (including smallholders that only raised fattening pigs) who had been identified and, among consenting participants, administered a structured questionnaire. We also asked participants if they could provide contact information of other actors with whom they traded pigs. Where possible, we contacted gatekeepers of these new villages and repeated the process between 1^st^ April and 8^th^ May 2023. A total of 23 villages were visited, resulting in interviews with five commercial farms and 182 smallholders. Interviews were predominantly conducted in the Laotian language, although some interviews involved the use of Chinese dialects with support from village leaders. The questionnaire collected information on (1) demographics of participants; (2) income sources and pig raising history; (3) number and type of pigs raised and housing condition; (4) cleaning, feeding, biosecurity and quarantine practices; (5) inward and onward pig movement in the past 12 months; and (6) poultry raising practices. Some data collected through the questionnaire were triangulated by field observations. The structured interviews took between 30 and 55 min. Data were entered into tablet computers preinstalled with Open Data Kit (ODK) software and dual‐language (Laotian and English) questionnaires and can be found at 10.5281/zenodo.15119504.

### 2.4. Slaughterhouse Sampling

We conducted slaughterhouse sampling between 31^st^ March and 29^th^ April 2023 at seven slaughterhouses in Oudomxay and Luang Namtha provinces. A total of 515 pigs, derived from 59 batches (where a batch is defined as a group of pigs delivered together at the slaughterhouse by a given farm or trader), were subjected to biological sampling. We collected 515 nasal swab samples, along with 313 whole blood samples and 319 serum samples. Samples were stored at −70°C (swabs) and −20°C (serum/blood) at the National Animal Health Laboratory (NAHL) in Vientiane. Viral RNA was extracted from swab samples using the QIAmp Viral RNA Mini Kit (Qiagen), and real‐time RT‐PCR was performed to detect the presence of IAV (M gene) using AgPath‐ID One‐Step RT‐PCR Kit (Thermo Fisher Scientific) at NAHL. Serum samples were subject to commercial ELISA–based assays (ID Screen, Innovative Diagnostic) to assess seroprevalence of IAV antibodies (indicating past exposure) among the sampled pigs.

### 2.5. Statistical Analyses

All data analyses were conducted using R statistical software (version 4.4.0) [16]. Descriptive analysis was carried out for the cross‐sectional data to define a pig producer typology and compare production and trading practices among producer types. A hierarchical Bayesian logistic regression was developed for the slaughterhouse data to evaluate risk factors associated with a higher IAV seropositivity while accounting for the effect of clustering within batches, following [17]. This analysis was not performed for the IAV active shedding status due to the small number of positive samples. A directed acyclic graph (DAG) was constructed (Figure S1) to describe the causal relationship between variables using the Dagitty R package [18]. A Cauchy distribution (location = 0 and scale = 10) was used as a weakly informative prior distribution for all exposure variables, and the default prior (student *t*(3,0,2.5)) was used for all intercepts. Posterior odds ratios of the exposure variables were estimated through the Hamilton Monte Carlo (HMC) method using Stan v2.26.1 through the brms package v2.20.1 by running three independent chains for at least 4000 iterations each and discarding the first 1000 samples as burn‐in. Model convergence was confirmed by checking Rhat values to be <1.01, large effective sample sizes, and trace plots for good mixing.

### 2.6. Within‐Farm IAV Transmission Modelling

An individual‐based mathematical disease simulation model was developed to estimate the duration of IAV persistence and immunity on different farm types. Given the paucity of commercial farms in northern Laos, we focused on modelling IAV transmission in smallholders. Individual pigs were partitioned into one of five mutually exclusive disease states: immune due to maternally derived antibody (M), susceptible (S), exposed (E), infectious (I), and immune after having recovered from infection (R). The disease status of each pig was tracked using a discrete‐time simulation with daily time steps. In an infected herd (i.e. a herd with at least one infectious animal), the force of infection *λ* for each susceptible individual in the same herd is described as:

λt=βItNt,

where *β*, *I*_*t*_, and *N*_*t*_ represented the transmission coefficient, the number of infected pigs, and the total number of pigs in the herd at a given day *t*. Pigs in E status moved to I status after a latent period of 2 days [19] and from I status to R status after 6 days [20]. Pigs in M status and R status became S status after an immune period that was randomly drawn from gamma distributions with mean durations of 70 days [20] and 180 days [21], respectively (scale = 3 for both parameter distributions). Transmission was assumed to be frequency‐dependent with *β* = 0.5, which was equivalent to a within‐herd basic reproduction number, *R*_0_, of 3 when the infectious period was 6 days [20, 21]. The model also tracked the production stage and events of individual pigs including mating, farrowing, weaning, and slaughtering. Relevant parameters were drawn from the questionnaire data. The detailed description of the within‐herd model can be found in the Supplementary document and elsewhere (https://github.com/arata-hidano/swIAV_transmission_model).

For each smallholder type except fattening herds, we simulated herds with different numbers of sows and boars based on the frequency distributions observed in the questionnaire data. The simulation was not conducted for fattening herds as the questionnaire data suggested they have a simple ’all‐in‐all‐out’ herd structure where a group of pigs of the same age remain in herds for a short duration. We simulated the disease transmission over 5 years, where the duration of IAV persistence and immunity was recorded. The persistence duration was defined as a period between the introduction of IAV (i.e. an individual becomes E status in herds without pigs in E or I status) and the elimination of IAV (i.e. an individual becomes R status in herds that no longer have pigs in E or I status). The immunity duration of a herd was defined as a period in which all pigs in the herds are in either M or R status. The simulation was carried out 500 times for each herd with a given type and herd size. Multivariable fractional polynomial regression models were developed to quantify the impact of the number of sows and boars (explanatory variables) on the simulated durations of persistence and immunity (outcome variables) by identifying the best functional form of explanatory variables using the R package mfp [22].

### 2.7. Between‐Farm IAV Transmission Modelling

We developed a between‐herd infectious disease modelling framework to simulate the spread of IAV among a simulated population of swine smallholders in northern Laos. This framework was a discrete‐time, spatially explicit, stochastic, agent‐based network model in which the epidemiological unit was a smallholder node. The model accounted for the duration of persistence and immunity for each farm type, informed by outputs of the within‐herd modelling; the variability in pig trading practices based on the cross‐sectional survey; and the inferred geographical distribution and spatial densities of smallholders.

The model adopted a susceptible‐infectious‐recovered‐susceptible (SIRS) structure. Smallholder nodes could transition from susceptible to infectious if they were in contact with an infectious node in a previous 1‐week timestep, from infectious to recovered after a defined infectious duration had elapsed and returned to susceptibility after a specified immunity duration had elapsed. Model details are described in the Supplementary document and elsewhere (https://github.com/wtm-leung/Laos-swIAV-transmission-model). In short, the transmission process through direct, indirect and spatial contact was modelled by simulating the pig farm population and pig trade in‐between in the study region.

### 2.8. Between‐Herd Model Scenarios

Simulations were performed using 18 transmissibility scenarios based on combinations of direct, pT(d); indirect, pT(i); and spatial, pT(s) transmission probabilities (Table S1). Infection was randomly seeded in nodes (*n* = 2) either of a defined smallholder type, in a high or low pig density region (defined as GLW3 cells with >1500 or ≤1500 pigs per cell, respectively), or else, totally at random – termed ‘total random seeding’. Based on combinations of 18 transmissibility and 9 seeding scenarios, 162 scenarios were run, each with 100 iterations, over a simulated time period of 2 years.

Our data indicated that smallholders were not directly connected to large commercial farms by trade. However, commercial farms might still play an important role for IAV transmission to nearby smallholders, such as through airborne and fomite spread [23]. Furthermore, persistent IAV infection can occur in large herds with high population turnover, as evidenced by field observation [24–26] and modelling studies [27]. We therefore also explored hypothetical scenarios involving persistent infections on large commercial farms. In these scenarios, we simulated a small number of large commercial breeding farms (*n* = 5), isolated from the pig trade network. These farms were randomly assigned spatial locations within high pig density areas and remained persistently infected for the entire simulation period.

### 2.9. Between‐Herd Model Outcomes

Due to the stochastic nature of the model, the size of simulated outbreaks varied across iterations of the same scenario. Outbreak size was plotted across all scenarios to identify, through visual inspection, an appropriate cut‐off for defining an epidemic. Two outcomes were computed. Epidemic probability was defined as the proportion of iterations generating an epidemic. This metric was used to indicate the propensity for onward transmission from different seed node types. Cumulative epidemic attack rate was calculated as the proportion of ever infected nodes at each time step and used as an indicator for actors’ susceptibility to infection after total random seeding. Due to the uncertainty in parameter values, we compared the qualitative differences among scenarios rather than focussing on the quantitative predictions.

### 2.10. Ethics

The study was approved by the LSHTM Institutional Review Board (IRB) (ref 22675) and Animal Welfare and Ethics Review Board (ref: 2021‐10), by the National Ethics Committee for Health Research in Laos, and the U.S. Navy Human Research Protection Program (HRPO.NAMRU2.2023.0001).

## 3. Results

### 3.1. Characteristics of Commercial Farms in Northern Laos

Five commercial farms were identified and interviewed in Oudomxay province, while none were identified in Luang Namtha province at the time of our visit (geographical location of the study cite is shown in Figure S2). Four commercial farms represented fattening production systems, and one was a farrow‐to‐finish system. Three fattening farms operated as contract farms, and the remaining two operated independently. The median herd size of commercial fattening farms was 910 (range 500–1200). The commercial farrow‐to‐finish farm kept 100 sows, 2 boars, 405 piglets, and 200 finishers at the time of the visit. The commercial farms had been in operation for between 3 and 7 years. Contract fattening farms received replacement piglets from their parent company twice a year. Details on the production cycle and husbandry practices of the farrow‐to‐finish commercial farm can be found in Box 1. Three out of four fattening commercial farms also kept poultry such as backyard chickens (range 40−100) and ducks (only 1 farm; *n* = 13). No commercial farms reported mass mortality among their poultry in the past 3 years. They buried dead poultry on the farm and mentioned that they never fed the carcass to pigs.

**Box 1. Description of the farrow‐to‐finish commercial farm in Oudomxay province.**
The farrow‐to‐finish commercial farm sometimes introduced replacement gilts from Thailand (the last time they introduced 60–80 gilts was in 2020). They did not sell piglets to other farms but provided boar semen for smallholders. This farm had two buildings: one for breeding and one for fattening production. Two to three sows farrowed each week (this was the maximum farrowing they could handle with their labour), and weaned piglets moved from the breeding building to the fattening building on day 78. Each sow farrowed twice a year, producing about 10 piglets each time. While they had boars, they implemented artificial insemination only (boars were kept to stimulate sows’ oestrous cycles). They sent around 20 finishers to the slaughterhouse every week. Sows were placed individually in a pen, whereas about 35 growers were kept in a single pen. Pens were cleaned every day using water and disinfectant.
They also kept poultry such as layers (*n* = 20), ducks (*n* = 4), and geese (the number was unknown). They did not purchase these poultry and produced the replacement on their own. Dead poultry were reported to be buried on the farm. These poultry as well as wild birds were able to contact directly with pigs. They implemented various biosecurity measures including boot dip and disinfection of vehicle wheels at the farm entrance, using disinfectants provided by their companies, personal protective equipment (PPE) among farm workers, and the restriction of access to pig pens. They vaccinated pigs against Aujeszky’s disease.

### 3.2. Characteristics of Smallholders in Northern Laos

Apart from ethnicity, demographics of interviewed smallholders in both provinces were similar (Table S2). The median duration smallholders had been raising pigs was relatively short (~3 years), although some had been raising pigs several decades. Many smallholders said they had only recently reintroduced pigs to their smallholding operations after pausing this activity due to the ASF outbreak, which was first confirmed in 2019 [28].

We then identified the typology of smallholders based on whether they provided boar service and their production characteristics (i.e. fattening, breeding, or both) in the past year, resulting in six main smallholder types: boar service provider (BSP), breeding smallholders with boar(s), breeding smallholders without boar(s), fattening smallholders, farrow‐to‐finish smallholders with boar(s), and farrow‐to‐finish smallholders without boar(s). There was only one fattening smallholder with boar(s); hence, this was combined with fattening smallholders without boar(s). The number of pigs raised in each smallholder type is shown in Table 1. The distribution of smallholder types was not statistically different between provinces (χ^2^ = 6.18, df = 5, and *p* = 0.29). However, smallholders in Oudomxay tended to keep more sows than those in Luang Namtha (Wilcoxon test *p* = 0.02). In terms of pig breed, exotic or crossbreed growers were kept only by five smallholders out of 54 which kept at least one grower pig. The median number of growers kept by these five smallholders was 19, which was significantly greater than that of growers kept by smallholders (value = 2) that had only local growers (Wilcoxon test *p*  < 0.01).

**Table 1:** Median and range of the number of pigs raised by each smallholder type in northern Laos.

Typology	Boar		Sow		Piglet/weaner		Grower/finisher	
Boar service provider (*n* = 12)	1	(0–2)	2	(0–9)	2.5	(0–36)	0	(0–3)
Breeding with boar (*n* = 17)	1	(1–2)	5	(0–37)	9	(0–70)	0	(0–4)
Breeding without boar (*n* = 16)	0	(0–0)	1	(1–4)	0	(0–6)	0	(0–1)
Fattening (*n* = 68)	0	(0–1)	0	(0–6)	0	(0–15)	0	(0–60)
Farrow‐to‐finish with boar (*n* = 24)	1	(1–3)	3	(1–35)	5	(0–58)	0	(0–21)
Farrow‐to‐finish without boar (*n* = 45)	0	(0–0)	1	(0–9)	1	(0–14)	0	(0–4)

We summarised key reproduction and production parameters that can affect within‐herd IAV dynamics. The litter size was significantly larger for breeding smallholders with boars (linear regression coefficient = 1.8, 95% confidence interval [CI]: 0.07–3.5, and *p* = 0.04) and farrow‐to‐finish with boars (coefficient = 1.9, 95%CI: 0.4–3.4, and *p* = 0.02), compared to that of farrow‐to‐finish without boars (Table S3). Farrow timing across smallholder type is shown in Figure S3. Many smallholders without boars belonged to groups where either most sows farrowed or did not farrow at all in the past 3 months, indicating that sows farrow in a cluster on these smallholder types. Most breeding and farrow‐to‐finish smallholders raised their own gilts, while some smallholders sourced gilts from smallholders in their own or other villages, or sometimes commercial farms (Table S4). Most smallholders with boars used their own boars only (Table S5). Some smallholders without boars claimed that they used their own boars, likely because they used to keep boars. Among smallholders who claimed they hired boars, most used only one specific BSP (Figure S4).

### 3.3. Characteristics of Traders and Slaughterhouse Users

Five slaughterhouse owners or managers (hereafter referred to as slaughterhouse people [SHP]) and 10 traders from four slaughterhouses were interviewed (total number of respondents = 15). No respondents traded poultry. The median duration of business operation was 9 years (range: 4–15). Most of the traders worked alone, although two traders belonged to a group of 6–8 traders. As shown in Table 2, respondents had purchased pigs on a median of 4 days (range 0–8 days) in the past month. Among four SHPs who provided data on trading volume, the median was 120 pigs (range: 12–564 pigs) purchased in the past 30 days, while traders purchased a median of 26 pigs (range: 3–150 pigs) in the past 30 days. In terms of the source of pigs, seven traders purchased only from commercial farms, whereas the remaining three traders purchased only from smallholders. Nine respondents transported live pigs, and among these, only one trader kept pigs at an intermediary location, usually for 4 days, before sending them to the final location. All nine respondents who transported live pigs used a single vehicle and transported a median of 10 (range: 5–40) pigs per trip. Two of these nine respondents used the same car to transport buffaloes and cattle. Five respondents said they delivered live pigs to multiple sites in a single trip, and six said they purchased live pigs from multiple sites in a single trip.

**Table 2:** Characteristics of traders and slaughterhouse users from four slaughterhouses in Northern Laos.

Variable	*n* ^1^	Median	Min	Max
Trading days^2^	14	4	0	8
Number of pigs purchased from each source type^3^				
Small household (<10 pigs)	5	5	2	80
Medium household (10–49 pigs)	4	4.5	1	72
Large household (50–99 pigs)	2	40.5	1	80
Small farm (100–399 pigs)	2	30	10	50
Medium farm (400–1999 pigs)	9	50	7	492
Large farm (2000~ pigs)	0	—	—	—
Days kept at slaughterhouse				
Average	13	4	3	7
Minimum	13	1	1	3
Maximum	13	7	5	10
Busy month and maximum number of pigs traded per day^4^				
January	13	5	1	300
February	2	180	60	300
April	5	5	3	8
May	1	2	—	—
June	1	2	—	—
November	11	4.5	1	60
December	11	5	1	60

^1^Number of respondents.

^2^Number of days that respondents were actively trading pigs in the past 30 days.

^3^Total number of pigs traded in the past 30 days from each source type.

^4^Number of respondents that selected given months as busiest. For a given month mentioned, the median and range of the maximum number of pigs traded per day were calculated among the respondents who selected the month.

### 3.4. Slaughterhouse Sampling

A total of 319 sera and 515 nasal swab samples were obtained from pigs and tested by ELISA and PCR, respectively. Overall, 100 sera (31.3%) tested seropositive for IAV antibodies, and seven swabs (1.4%) tested positive for detectable viral RNA. Data on individual pig‐ and batch‐level variables were available for 305 sera and 496 nasal swab samples from 57 batches for further analyses. In terms of geographic origin, 112 pigs (22.6%) from nine batches (15.8%) were sourced from another province, including eight batches from Vientiane and 1 from Luang Prabang. Only 10 serum samples were from smallholder pigs, of which three were local pig breeds. Most serum samples originating from commercial farms were from exotic pig breeds (*n* = 116, 84.1%). Farm‐type origin was unknown for over half (*n* = 157) of the serum samples, with respondents reporting only that they acquired these pigs from traders. Of these 157 pigs, 154 were exotic pig breed (98.1%) and three were local pig breed.

IAV positivity varied across pig and batch‐level factors (Table 3). The majority of samples were from exotic breed pigs, of which 33.6% and 1.2% tested positive by ELISA and PCR, respectively. On the other hand, only six sera (2.0%) and 48 swabs (9.7%) were from local breed pigs, which tested negative for both ELISA and PCR. The small sample size for local breed pigs reflects how slaughterhouses in northern Laos primarily process pigs from semicommercial/commercial farms and relatively large smallholders, whereas pigs from more rural smallholders (where local breeds are raised) are often slaughtered at informal killing points or by households.

**Table 3:** Influenza A virus seropositivity (ELISA) and positivity (PCR) at pig and batch levels.

Variable	Sample size	Positive	(%)	Sample size	Positive	(%)
Individual pig level	ELISA (*n* = 305)		—	PCR (*n* = 496)		—
Sex	—	—	—	—	—	—
Male	136	43	31.6	205	4	2.0
Female	169	57	33.7	291	3	1.0
Breed						
Exotic	271	91	33.6	408	5	1.2
Cross	28	9	32.1	40	2	5.0
Local	6	0	0	48	0	0.0
Province						
Oudomxay	241	80	33.2	427	2	0.5
Luang Namtha	64	20	31.2	69	5	7.2
Source						
Large commercial	40	13	32.5	49	2	4.1
Medium commercial	98	40	40.8	106	1	0.9
Medium smallholder	0	0	0	4	0	0.0
Small smallholder	10	6	60	49	0	0.0
Trader	157	41	26.1	288	4	1.4
Batch level	ELISA (*n* = 57)		—	PCR (*n* = 57)		—
Sampling province						
Oudomxay	50	30	60	50	2	4.0
Luang Namtha	7	5	71.4	7	2	28.6
Source						
Large commercial	5	4	80	5	1	20
Medium commercial	12	11	91.7	12	1	8.3
Medium smallholder	1	0	0	1	0	1
Small smallholder	9	3	33.3	9	0	0
Trader	30	17	56.7	30	2	6.7
Movement pattern						
Within district	34	16	47.1	34	1	2.9
Within province	13	11	84.6	13	1	7.7
Between province	10	8	80.0	10	2	20.0

Table 4 shows the results of multivariable Bayesian hierarchical logistic regression models to estimate the direct effect of variables of interest while accounting for the clustering effect of batches. For each exposure variable, model covariate selection was based on the minimally sufficient adjustment set indicated by the DAG (Figure S1). Pigs from smallholders were significantly more likely to test seropositive compared to those from commercial farms; however, with only 10 serum samples from smallholder pigs, confidence intervals were wide (adjusted odds ratio [aOR] 101.60 and 95% credible interval [CI]: 1.48–1.20 × 10^4^). Local pigs were less likely to test seropositive compared to exotic breed pigs, with a wide CI due to the small sample size. Pigs that were imported from external provinces were significantly more likely to test seropositive than those sourced within the district (aOR 5.07 and 95%CI: 1.07–25.51) after adjusting for potential confounding effects. Batch size and sex were not significantly associated with seropositivity.

**Table 4:** Multivariable analysis of associations with influenza A virus seropositivity (ELISA) among pigs using Bayesian hierarchical logistic regression modelling.

Variable	Model^1^	Level	Adjusted odds ratio	95% Credible interval	
				Lower	Upper
Source farm type	1	Commercial farm	Reference		
		Smallholder	101.60	1.48	1.20E + 04
		Trader	0.76	0.06	10.52

Breed	1	Exotic	Reference		
		Cross	0.30	0.03	3.02
		Local	2.87E‐12	3.02E‐49	7.94E‐03

Batch size	2	1–7	Reference		
		8–12	0.90	0.28	2.82
		13–24	0.98	0.31	3.07
		25+	0.78	0.2	2.99

Origin province	3	Oudomxay	Reference		
		Luang Namtha	4.10E‐09	4.80E‐36	1.13E + 02
		Luang Prabang	3.70	1.25E‐06	3.43E + 07
		Vientiane (capital)	0.80	8.20E‐06	3.70E + 04
		Vientiane (province)	1.40	1.31E‐05	1.61E + 05

Transport distance	4	Within district	Reference		
		Within province	1.96	0.17	2.19
		Between province	5.07	1.07	25.51

Sex	5	Female	Reference		
		Male	0.88	0.51	1.51

^1^Variables included in the model: Model 1: source farm type, breed, batch size, slaughterhouse ID, and transport distance; Model 2: source farm type, breed, and batch size; Model 3: source farm type, origin province, breed, slaughterhouse ID, and transport distance; Model 4: source farm type, breed, slaughterhouse ID, and transport distance; Model 5: sex.

### 3.5. Within‐Herd Disease Modelling

The distributions of the persistence of IAV infection and immunity within BSP herds, derived from outputs of within‐herd model simulations, are shown in Figure 1A,B. Persistence of IAV within smallholder herds was generally short regardless of the number of pigs and farm types, with a median of 9 days. On rare occasions, however, persistence was prolonged over several weeks. For example, in 5% of simulations, IAV persisted within herds for >34 days, up to a maximum of 64 days for BSP with nine sows and two boars. As shown in Figure 1C, the addition of sows tended to increase the average persistence duration regardless of the farm type (Table S6). However, this trend became more complex once the number of sows exceeded 10 (Figure S5), with median duration starting to decrease (reflecting an intensified force of infection in larger, fully susceptible herds) but the maximum duration continuing to increase. In terms of immunity, the median duration was 152 days for BSP with one sow and one boar. The addition of sows decreased the immune duration in all farm types (Figure 1B; Table S6). A similar trend was observed with the addition of boars, except in BSP where the impact was negligible (Table S6).

Figure 1Results of within‐herd IAV transmission models. Distributions of (A) IAV persistence; (B) immune durations in boar service providers (BSP) stratified by the number of sows and boars, with median values depicted by black circles; and (C) predicted IAV persistence durations for each farm type (the number of boars was set to 1) using a multivariable fractional polynomial regression. Shaded areas indicate 95% confidence intervals.

**Figure:**
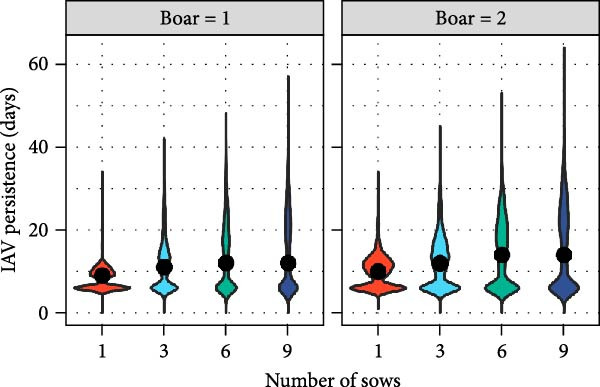
(A)

**Figure:**
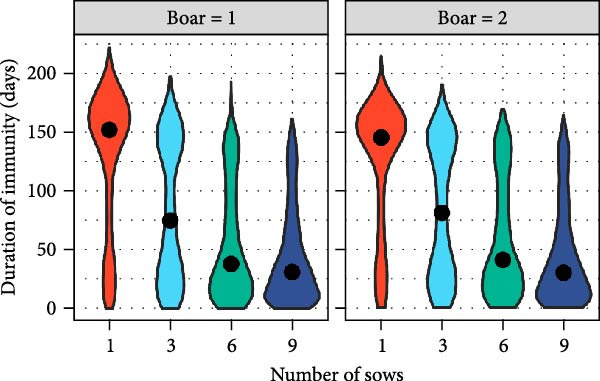
(B)

**Figure:**
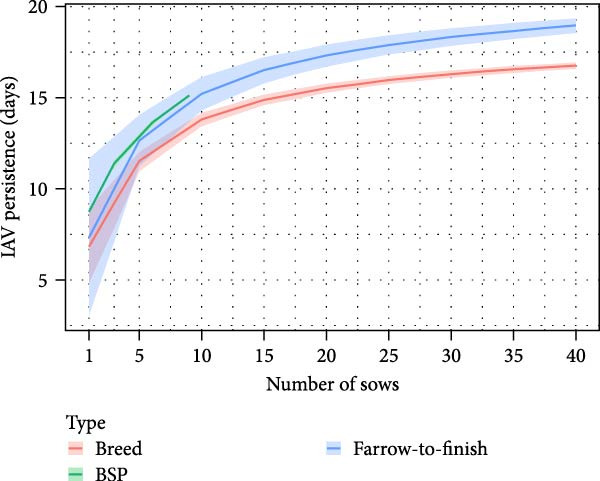
(C)

### 3.6. Network Calibration and Node Removal

The simulated swine trade network was well calibrated to the observed distributions of in‐ and out‐degree (Figure S6), mixing patterns among smallholder types (Figure S7), and the distribution of geolocations in which trade partners were located (Table S7). However, the simulated network generated BSP with a greater proportion of isolated and low‐degree nodes than was observed.

The simulated 1‐year pig trade network had a greater proportion of isolated nodes (i.e. smallholders not engaged in trading) in comparison to a random graph with equivalent density (Table S8). The 1‐year pig trade network had 120 weakly connected components (WCC), defined here as sets of at least two nodes which were reachable from each other (irrespective of edge directionality). Average geodesics (i.e. shortest path lengths between pairs of nodes) were also shorter in the pig trade network indicating a potential for relatively rapid disease transmission. Converting the 1‐year network to a 1‐week network generated much sparser networks (i.e. a large fraction of isolates) and very small WCC, indicating a limited potential for disease transmission on a static 1‐week network.

We explored the robustness of these networks by computing the size of the WCC following targeted node removal. Targeting nodes with the highest network metric values (i.e. in‐degree, out‐degree, betweenness, or closeness) was more effective in fragmenting the simulated swine trade network than random node removal, with targeting by out‐degree being most effective (Figure 2A). Removal of 6% of the highest out‐degree farms was sufficient to reduce the component size by 96%. When targeting by smallholder type, only removal of BSP and breeding farms with boars were more effective strategies than random node removal (Figure 2A). This finding corresponds with the relatively high in‐ and out‐degree of these smallholder types (Figure S6). Removal of all breeding farms not keeping boars was sufficient to reduce the component size by ~45%. Removing BSP was comparatively less effective on account of the relatively smaller impact of removing these actors and the small number of BSP within the network.

Figure 2Component size following targeted node removal in the simulated 1‐year swine trade network (A) and spatial network (B). Targeting nodes by network metrics removed nodes sequentially, starting with nodes with the highest values. Targeting nodes by actor types (A) and pig density regions (B) was performed randomly. For actors and pig density regions, nodes were removed until no nodes of that type remained resulting in lines of varying length.

**Figure:**
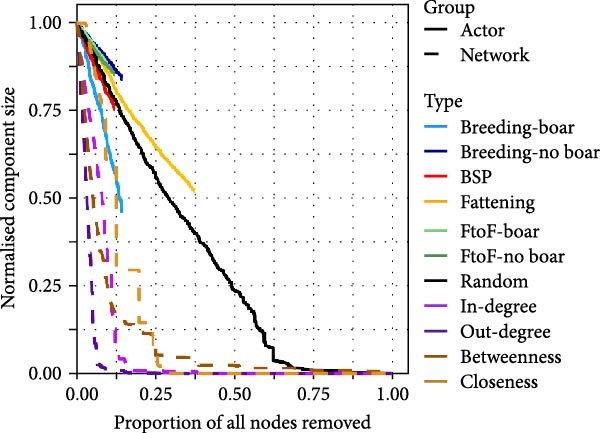
(A)

**Figure:**
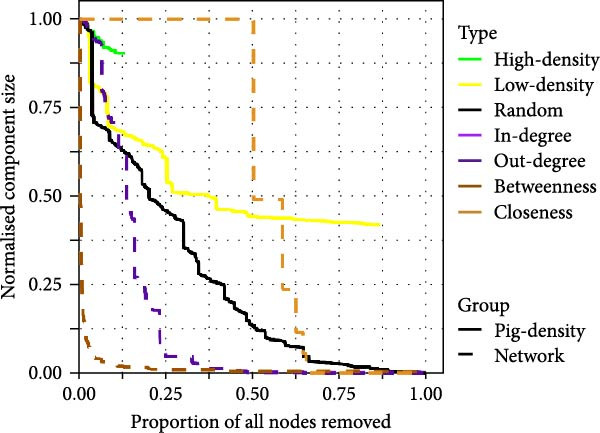
(B)

The most efficient means of fragmentating the spatial network was by targeting nodes with the highest betweenness or out‐degree. All other methods were less efficient than random node removal (Figure 2A). Removal of nodes in high pig density areas had relatively little impact compared to removal of nodes in low‐density areas which can be explained by the presence of numerous alternative paths between nodes in the former.

### 3.7. Between‐Herd Infectious Disease Modelling

#### 3.7.1. General Epidemic Features

An ‘epidemic’ was defined as ≥65 infected nodes based on the distributions of outbreak size (Figure S8). Most simulations (81%) generated outbreaks under this threshold. Outbreaks were self‐limiting across the range of scenarios tested that is the infection process ended before the simulation end.

Epidemics never occurred when the probability of transmission from spatial contact, pT(s), was below 0.2 (the maximum value tested). This can be explained by the low density of the direct (and indirect), 1‐week, pig trade networks—itself a function of the infrequent trades observed among smallholders—and the short durations of node‐level persistence (Figure S9). All results presented are therefore for pT(s) = 0.2 only.

#### 3.7.2. Epidemic Probability and Attack Rate

The smallholder type in which the infection was seeded appeared to be a poor predictor of epidemic probability (Figure 3A). While the probability of an epidemic varied between different types of seed node for a given transmissibility scenario, patterns were not consistent across transmissibility scenarios. Across the range of transmissibility scenarios tested, final epidemic attack rate (i.e. the proportion of nodes ever infected) was greatest in BSP, followed by breeding farms, and was lowest in farrow‐to‐finish farms. Despite these differences, there was substantial overlap in the 5^th^–95^th^ percentiles of attack rates among simulation iterations (Figure 3B).

Figure 3Estimated epidemic probability and cumulative epidemic attack rate: (A) epidemic probability after seeding in defined smallholder types, (B) attack rate by smallholder type in random seeding scenario, (C) epidemic probability stratified by seeding scenarios where Rand indicates random seeding scenario, and (D) attack rate high‐ and low‐density regions in random seeding scenario. Solid lines show median cumulative epidemic attack rate; dashed lines show inter‐95‐percentiles across 100 iterations. Results of pT(i) = 0.1 were similar to those of pT(i) = 0.2 and not shown.

**Figure:**
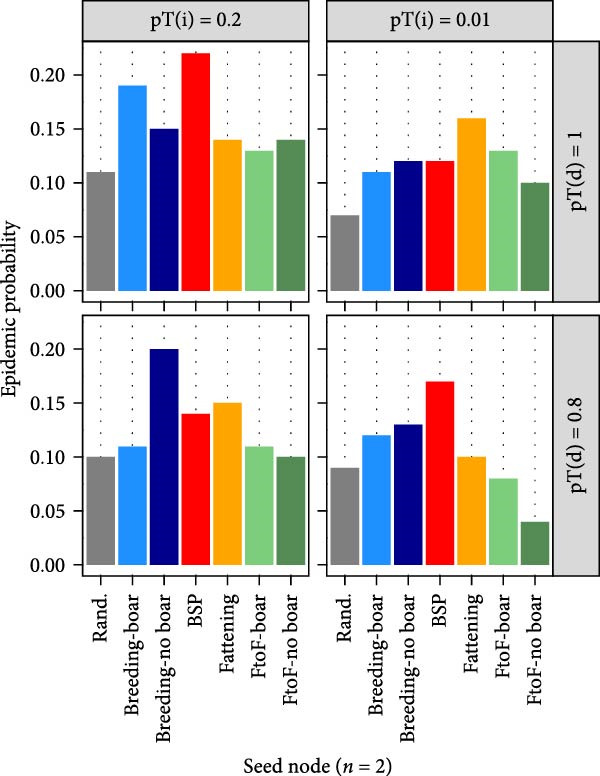
(A)

**Figure:**
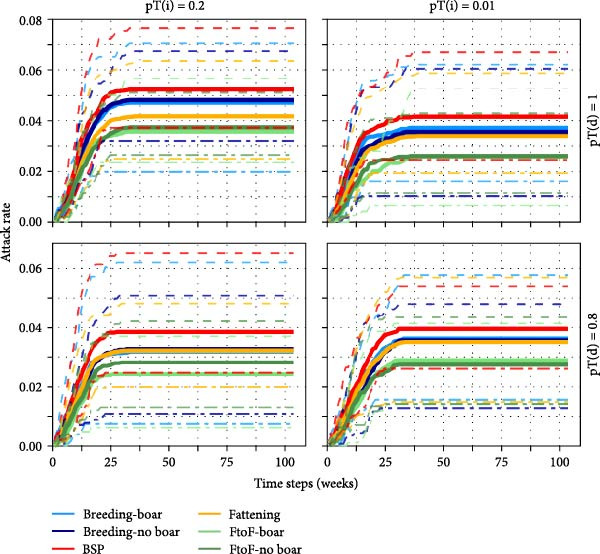
(B)

**Figure:**
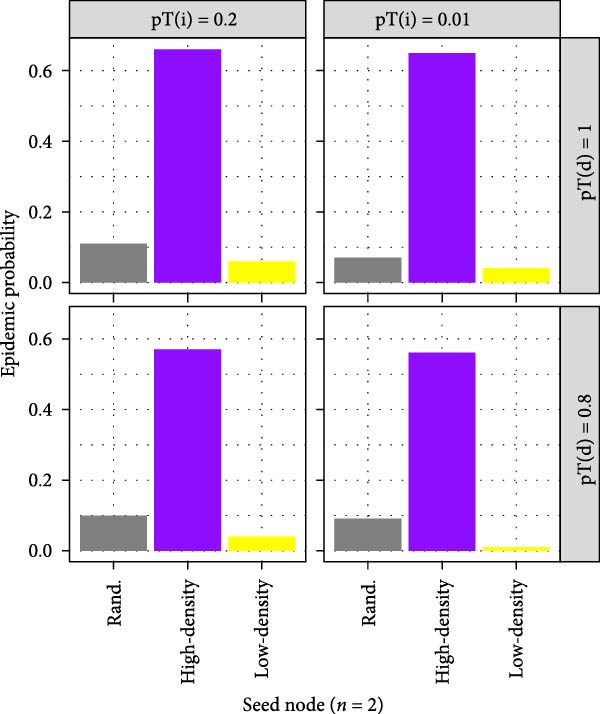
(C)

**Figure:**
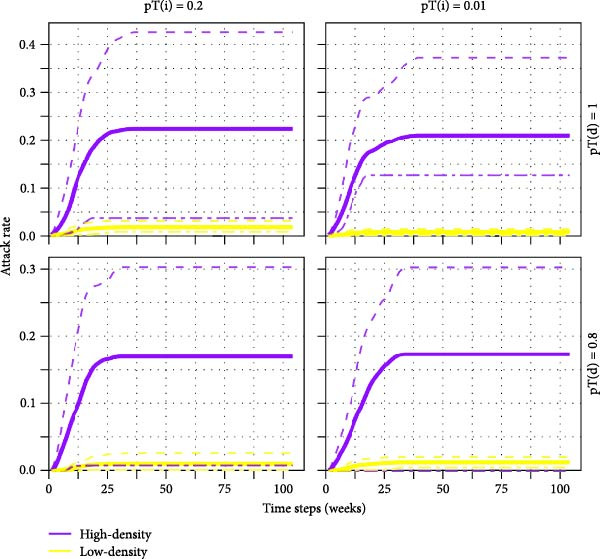
(D)

Seeding of infection in smallholders located in high pig density grid cells generated markedly greater epidemic probabilities compared to seeding in those in low‐density areas (Figure 3C). Smallholders located in high pig density regions also experienced much greater median epidemic attack rates throughout the course of simulations; however, some overlap in attack rates was again observed among simulation iterations (Figure 3D).

#### 3.7.3. Proportion of Infectious and Recovered Nodes

**Figure 4:**
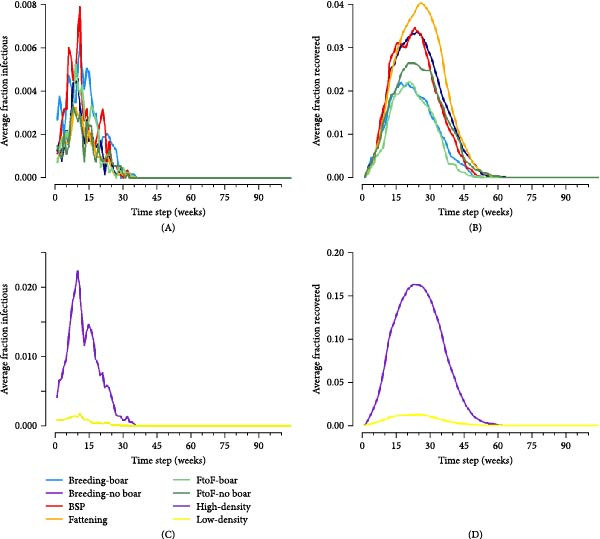
Infectious disease dynamics by actor and region. The mean proportion of infectious (A and C) and recovered (B and D) nodes over the course of the simulation by smallholder type (A and B) and pig density region (C and D). Results are shown here for the highest transmissibility scenario following random actor seeding (see supplementary for the full range of transmissibility scenarios).

No clear patterns in node‐level infection prevalence by smallholder type were observed (Figure 4A and Figures S10 and S11). However, node‐level seroprevalence (i.e. the fraction of recovered nodes) reached the highest level and remained highest for the longest period in fattening farms (Figure 4B). This observation was consistent between transmissibility scenarios (Figures S10 and S11) and can be explained by the relatively high duration of IAV persistence in an average fattening farm (Figure S9).

#### 3.7.4. Scenarios of Persistently Infected Commercial Farms

**Figure 5:**
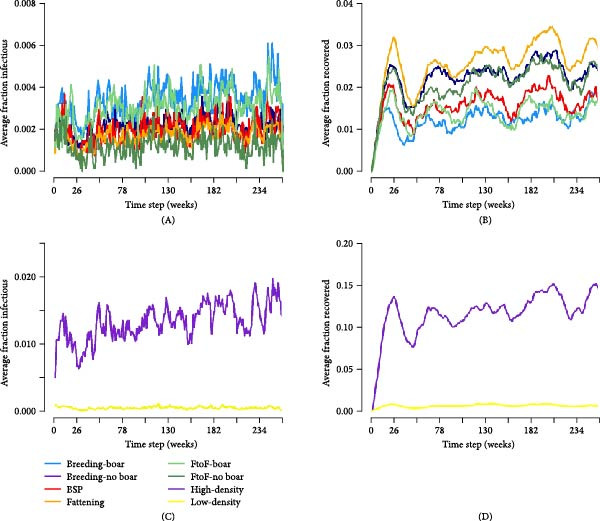
Infectious disease dynamics by smallholder type and pig density region in scenarios with persistently infected large commercial farms (*n* = 5). The mean proportion of infectious (A and C) and recovered (B and D) nodes over the course of the simulation by smallholder type (A and B) and pig density region (C and D). Results are shown here for the highest transmissibility scenario following random actor seeding.

The inclusion of five persistently infected farms within the simulated population of nodes was sufficient to reach endemic status, with the infection process sustained throughout the course of the simulation (Figure 5). In comparison to scenarios without large commercial farms, variation in infection prevalence was clearer among smallholder types, albeit only slightly, with node‐level prevalence (i.e. proportion of herds infected) highest among breeding farms throughout the course of the simulation (Figure 5). The relationship between farm type and epidemic probability or attack rate remained unclear (Figure S12A). However, the epidemic attack rate continued to increase throughout the course of the simulation indicating continual infection of previously uninfected farms (Figure S12B).

## 4. Discussion

Using a combination of pig sampling at slaughterhouses, cross‐sectional surveys among pig producers and exchangers, and mathematical modelling of disease transmission, this study represents, to our knowledge, one of the first studies to investigate IAV risk in relation to pig production and trading systems in Laos. Here, we summarise key findings and their implications for disease surveillance and control strategies, discuss some key limitations and identify priorities for further research.

The overall IAV prevalence (1.4%) and seroprevalence (31.3%) observed among pigs at slaughterhouses in northern Laos is comparable to ranges observed in other settings [29, 30]. Although this was a cross‐sectional study, these values may suggest endemic circulation of IAV among pigs within our study region. We note, however, that most of the pigs sampled were exotic breeds, and many were reported to have originated from commercial farms, despite relatively few commercial farms identified within our study area. We also identified that pigs were brought from as far as Vientiane province to meet the demand. This reflects the types of farm and pig breeds typically supplying slaughterhouses in northern Laos, while local breed pigs are often slaughtered at informal killing points in communities. We also observed that most smallholders had recently resumed pig production while keeping a relatively small number of pigs, which may be further driving pig movement from commercial farms in other provinces. The attitude towards pig raising among interviewed participants was, however, polarised: While many smallholders expressed concerns about losing pigs again due to outbreaks of serious diseases such as ASF, some considered the reduced supply of pigs in the area as an opportunity and had rapidly expanded their herds. These expanding herds kept a much greater number of pigs compared to what was previously reported [31, 32]. Our survey findings, when compared with those of previous reports, also suggest that there may have been some improvements in pig management practices. For instance, one study conducted in five provinces in northern Laos reported that the period between farrowing and the next mating was about 70 days [31], whereas our study identified many participants mated sows in their first heat after farrowing (21 days). This improvement was partly due to the adoption of exotic or cross‐breed boars that have improved productivity. Indeed, about 20% of farrow‐to‐finish and breeding smallholders with boars kept crossbred or exotic boars in this study.

Interestingly, in our multivariable analyses, seropositivity was significantly higher among pigs originating from other provinces, implicating interprovincial pig movements as an important pathway for geographic spread and the introduction of swIAVs into our study area. On the other hand, this also means that these epidemiological data from slaughterhouses may not be entirely representative of pig populations raised by local, small‐scale producers in our study area. Similarly, after adjusting for other variables, seropositivity was higher among pigs reportedly originating from smallholders compared with commercial farms. While the small sample size (and inherent wide credible intervals for some regression results) calls for careful interpretation, this result contrasts with studies in other settings in SEA, where IAV prevalence and seroprevalence are typically reported to be higher in commercial farms [17, 29]. However, most of our serum samples from smallholders were obtained from exotic or cross‐breed pigs; this is in contrast to the finding of our cross‐sectional survey that most smallholders in this region (except for some larger‐scale units) tended to keep local pigs. We therefore speculate these smallholders’ samples were from large smallholders or even semicommercial farms. Indeed, we found significantly lower seropositivity among local breed pigs than exotic breeds after adjusting for other factors. They together may suggest that future herd expansions, coupled with the adoption of exotic breeds, may intensify IAV circulation in this region. Although this hypothesis is also supported by our modelling study as we discuss below, a larger epidemiological study sampling more from local breeds in smallholders is warranted. The relatively long duration for which pigs are kept at slaughterhouses (up to 10 days) also requires attention. In our study area, due to the relatively small human population, only a small number of finishers were required daily to meet local pork demand. Yet, both fattening commercial farms and smallholders in the area adopt an all‐in‐all‐out system, where fattening pigs are introduced and sold as a group. Slaughterhouses in the area therefore received large batches of pigs which were slaughtered over a week, presenting opportunities for transmission of various diseases, including IAV, at slaughterhouses [17]. This may have affected the seropositivity and IAV shedding status of pigs sampled in this study. Larger, longitudinal studies are needed to comprehensively characterise IAV epidemiology among swine in northern Laos. Nevertheless, our findings provide valuable preliminary evidence from an understudied setting, which can inform the hypotheses and design of future studies.

Our modelling analyses focused on IAV transmission within and between smallholder herds. This was due to the predominance of smaller‐scale production systems in our study area (and thus also the availability of data on their production and trading practices in our cross‐sectional survey). Our within‐farm simulations of IAV transmission estimated that IAV transmission could typically persist within smallholder herds for ~1–3 weeks following virus introduction. However, there was notable variation both within and across different smallholder types, with longer persistence generally observed in BSPs and in breeding smallholders keeping their own boars, and persistence increasing with numbers of boars and sows kept.

For the between‐herd simulations of IAV transmission, we developed a model framework which allows for transmission via pig trading movements, as well as spatial transmission among geographically proximal smallholders independent of trading activities. Through our reconstruction of pig movement networks, while further model validation is needed, we illustrated how configuration algorithms represent a useful tool for simulating complete, ‘socio‐centric’ networks based on ego‐centric network data from a sample of nodes within a given study area. Our evaluations of the impact of ‘targeted node removal’ on network connectivity suggest that preventative disease control measures aiming to fragment the trade network are more effective when targeted among farms conducting the most trade, especially those with the largest numbers of recipients. While identifying farms with high out‐degree might be challenging in practice, targeting farms by type—specifically BSPs and breeding smallholders with boars—could serve as a simpler alternative.

Despite the presence of large component sizes in the 1‐year networks, these were considerably reduced in the scaled‐down 1‐week networks, limiting the potential for transmission of diseases which persist for short durations within a herd. This was illustrated in our between‐herd IAV transmission modelling, which suggested that contact networks dependent on pig trading between smallholders alone may not be sufficient to sustain transmission in our study setting. Epidemics only occurred in our between‐herd simulations when the probability of ‘spatial transmission’ (independent of pig movements) between geographically proximate smallholders was relatively high, suggesting that nontrade‐related contact may play an important role in IAV transmission between smallholders. We further found that larger and more sustained outbreaks occurred when infection was seeded in areas with higher densities of smallholders and that smallholders in high pig density areas experienced greater epidemic attack rates and reached higher serological and virological prevalence than those in low‐density areas. These findings are consistent with field observations in other settings which have found that pig density within a geographical area is an important risk factor for influenza (sero)‐positivity [33, 34]. Together, this highlights the potential value of targeting regions with high pig densities for influenza surveillance activities in Laos.

Even when accounting for spatial connectivity between smallholders, simulated epidemics were self‐limiting with no sustained or endemic state reached. Interestingly, however, we found that a small number of persistently infected large commercial farms could lead to sustained farm‐level infection prevalence among smallholders. While these scenarios were hypothetical and made a range of assumptions—for example, about the potential for persistence within large farms in Laos and the potential for IAV transmission from large farms to proximal smallholders—these assumptions can be valid. We found that most of the five commercial pig farms identified in our study area were fattening farms, which regularly source large supplies of pigs from their parent companies outside the province and can serve as a source of infection.

Overall, our data highlight the utility of slaughterhouses as relatively feasible and cost‐effective ‘sentinel’ sites for surveillance and monitoring of swIAVs with reasonably broad geographic coverage, particularly among pigs from commercial farms which can be difficult to access directly. Our findings are in line with the well‐established link between livestock intensification and evolving disease risks [35–38] and underscore the importance of continual monitoring of the IAV risk landscape as the swine sector intensifies in Laos.

## 5. Conclusion

Using a combination of slaughterhouse sampling, cross‐sectional surveys to understand pig producers’ practices, and mathematical modelling, this study indicated the heterogeneous risk of swIAV transmission across different types of pig producers in northern Laos. The modelling study suggested that a larger herd size may contribute to the longer IAV persistence, and the sustained IAV transmission between farms is unlikely in the region without the presence of farms persistently infected with IAV. With the intensification of the swine sector in Laos, continual monitoring of IAV risk using a relatively convenient approach such as slaughterhouse sampling is crucial, yet such results should be carefully interpreted by incorporating regional pig movement information.

## Disclosure

Jose A. Garcia‐Rivera (LCDR, USN, MSC), Anca Selariu (LCDR, USN, MSC), Robert Hontz (LCDR, USN, MSC), and Andrew G. Letizia (CAPT, USN, MC) are military service members. This work was prepared as part of their official duties. Title 17, U.S.C., §105 provides that copyright protection under this title is not available for any work of the U.S. Government. Title 17, U.S.C., §101 defines a U.S. Government work as a work prepared by a military service member or employee of the U.S. Government as part of that person’s official duties. The views expressed in the article are those of the authors and do not necessarily express the official policy and position of the US Navy, the Department of Defense, the US government, or any of the other institutions affiliated with any of the authors

## Conflicts of Interest

The authors declare no conflicts of interest.

## Author Contributions

Arato Hidano and William T. M. Leung contributed equally to this work.

## Funding

This work was funded by the Armed Forces Health Surveillance Division (AFHSD) Global Emerging Infections Surveillance (GEIS) Branch, ProMIS ID P0089_23_N2.

## Supporting Information

Additional supporting information can be found online in the Supporting Information section.

## Supporting information


**Supporting Information** Table S1. Transmission parameters for between‐herd influenza A virus (IAV) modelling estimated by within‐herd model. Table S2. Demographics of smallholders in Oudomxay and Luang Namtha province. Table S3. Distribution of litter sizes across smallholder type in northern Laos. Table S4. Distribution of the mode of replacing sows across smallholder type in northern Laos. Table S5. Distribution of the mode of boar usage across smallholder type in northern Laos. Table S6. Results of multivariable fractional polynomial regressions for the persistence and immune durations across smallholder type. Table S7. Comparison of distribution of locations in which trade partners were located as observed in the empirical data and in the simulated swine trade network. Table S8. Network statistics of simulated networks and their equivalent Erdős–Rényi random graphs with the same number of nodes and edges. Figure S1. Directed acyclic graph for the assumed causal relationship between explanatory variables and the outcome (infection on farm), which was approximated by the ELISA status. Figure S2. Map of the study site. Figure S3. Distributions of the proportion of sows that farrowed in the past 3 months stratified by smallholder type in northern Laos. Figure S4. Number of different boar service providers (BSPs) used by smallholders, who hired boars, in the past 1 year in northern Laos. Figure S5. Distributions of the simulated persistence and immune duration across smallholder type. Figure S6. Comparison of distributions of in‐ and out‐degree by smallholder type. Figure S7. Comparison of mixing matrices by smallholder type. Figure S8. Violin plot of the distribution of epidemic sizes following seeding in different smallholder types and pig density regions. Figure S9. Distributions of duration of node‐level persistence (A) and immunity (B) of the simulated population of nodes. Figure S10. Infectious disease dynamics by actor. Figure S11. Infectious disease dynamics by region. Figure S12. Scenarios of persistently infected large commercial farms.

## Data Availability

The data that support the findings of this study are available from the corresponding author upon reasonable request.
