# Supplementary material for: Epidemiology and Risk Modelling of Influenza A Virus Within and Between Pig Herds in Northern Lao PDR
Source: Transbound Emerg Dis. 2026 Jan 23;2026:2407533. doi: 10.1155/tbed/2407533 (PMC12829471; doi:10.1155/tbed/2407533)
Supplement: Supplementary file 1 — Supporting Information Table S1. Transmission parameters for between‐herd influenza A virus (IAV) modelling estimated by within‐herd model. Table S2. Demographics of smallholders in Oudomxay and Luang Namtha province. Table S3. Distribution of litter sizes across smallholder type in northern Laos. Table S4. Distribution of the mode of replacing sows across smallholder type in northern Laos. Table S5. Distribution of the mode of boar usage across smallholder type in northern Laos. Table S6. Results of multivariable fractional polynomial regressions for the persistence and immune durations across smallholder type. Table S7. Comparison of distribution of locations in which trade partners were located as observed in the empirical data and in the simulated swine trade network. Table S8. Network statistics of simulated networks and their equivalent Erdős–Rényi random graphs with the same number of nodes and edges. Figure S1. Directed acyclic graph for the assumed causal relationship between explanatory variables and the outcome (infection on farm), which was approximated by the ELISA status. Figure S2. Map of the study site. Figure S3. Distributions of the proportion of sows that farrowed in the past 3 months stratified by smallholder type in northern Laos. Figure S4. Number of different boar service providers (BSPs) used by smallholders, who hired boars, in the past 1 year in northern Laos. Figure S5. Distributions of the simulated persistence and immune duration across smallholder type. Figure S6. Comparison of distributions of in‐ and out‐degree by smallholder type. Figure S7. Comparison of mixing matrices by smallholder type. Figure S8. Violin plot of the distribution of epidemic sizes following seeding in different smallholder types and pig density regions. Figure S9. Distributions of duration of node‐level persistence (A) and immunity (B) of the simulated population of nodes. Figure S10. Infectious disease dynamics by actor. Figure S11. Infectious disease dynamics b [file TBED-2026-2407533-s001.zip › RISNIP_draft_Supplement_modelling_v5_Nov2025_revised.docx]

Supplementary Materials

# Epidemiology and risk modelling of Influenza A virus within and between pig herds in northern Lao PDR

Within-farm IAV transmission modelling

*Transition between production and infection stages*

Newly born piglets were weaned at 28 days old, when weaned piglets enter into a growing stage (28 days), after which they spent 84 days before being culled at 4-5 months old as finishers. Some growers became replacement sows (gilts), instead of finishers, and produced their first batch of piglets at 11 months old. Sows were mated 21 days after farrowing, and if they successfully became pregnant, they farrowed after 111 to 120 days. Piglets born from sows that were in R status were placed in M status with loss of immunity after a period drawn from the distribution specified above; piglets from sows in other statuses had S status. Each sow was culled after they had farrowed 8 times and completed the weaning process. When sows were culled, replacement gilts were selected among growers if they were present within herd; otherwise, the replacements were introduced from external farms. We assumed that gilts introduced from other herds were at E status with a probability of 0.1 (equivalent to prevalence of 0.1, accounting for a higher chance of these pigs being exposed (Garrido-Mantilla et al., 2021)) and otherwise at S status. In herds with boars, mating was done using their own boars, whereas external boars were hired in herds that did not have boars. Hired boars were at I status with a probability of 0.03 (equivalent to an infection prevalence of 3% (Zeller et al., 2023)), which infected the mated sows if they were in S status.

Network characteristics

A large weakly connected components (WCC) of 2449 nodes was present – representing the maximum extent of disease spread on a static network of this structure. Strongly connected components (SCC) are subgraphs in which all nodes are reachable from one another while taking into consideration edge directionality. More SCCs were present than in the equivalent random graph. The simulated spatial network was, as expected, lattice-like in structure with high clustering and long average geodesics relative to an equivalent random graph.

Between-farm IAV transmission modelling

*Transmission process*

Influenza transmission among pig herds can occur via direct contact (Torremorell et al., 2012), indirect contact through fomites (Allerson et al., 2013; Desrosiers, 2021; Poljak et al., 2008; Thompson and Bennett, 2017), or via “spatial” contact e.g. via airborne spread (Corzo et al., 2013), pig slurry (Desrosiers et al., 2004), or human population movements. In the model, transmission could occur via all of these processes. Direct contact could occur via pig movements from a supplier to a recipient node (*i to j*). Indirect contact could occur from a recipient to a supplier (*j to i*) based on an assumption of shared equipment or personnel (i.e. fomites). In addition, spatial contact among smallholders within relatively close proximity was incorporated to allow for transmission between nodes independently of pig trading activities. Corzo et al. (2013) found that IAV RNA could be detected in air sampled up to 2.1km away from large farms (i.e. keeping approximately 1,000-2,000 pigs). However, the likelihood of transmission via this route is sensitive to environmental conditions such as wind direction and temperature, and strain-dependent variation in transmission via this route (Hu et al., 2023). Moreover, given the relatively small size of pig herds in smallholder settings, we used a conservative threshold distance of 1km.

*Experimental population simulation*

We simulated a population of smallholders that matched the observed characteristics of the sampled smallholders. The population distribution of smallholder types was assumed equivalent to the sampled distributions. Each smallholder node was assigned a number of sows and boars by randomly sampling from the relevant distributions for each actor type.

Smallholders were assigned spatial locations within the study area. Since we did not know the exact locations of smallholders, we used Gridded Livestock of the World data (Gilbert et al., 2018) which predicts livestock densities at a resolution of ~1km square grid cells based on census data (Figure S2A). We used predictions from the ‘dasymetric model’ which estimates livestock densities per cell using a Random Forests model. Given that only 3% of pigs are kept by commercial farms (Ministry of Agriculture, Forestry and Fisheries Loas, 2019), for simplicity, the contribution of these farms to the total pig population was ignored. The number of pigs per cell was then converted to smallholders per cell by dividing by a mean herd size of 9.3 pigs based on our survey data. Within each cell, *n* smallholders were randomly assigned a point location. This process did not account for any spatial clustering of smallholders within a grid cell nor any heterogeneity in the spatial distribution of actors, for which data were unavailable. Based on these results, we calculated that there are 30,219 smallholders in the study area. To facilitate computational feasibility of the agent-based modelling simulation, we instead used a scaled down population of 4,000 nodes (~13% of nodes) within an area of ~90km^2^, selected to cover a region bordering two provinces and including high and low pig density regions (Figure S2B).

*Pig trade (direct contact) and fomite (indirect contact) network simulation*

Swine trade data collected via network surveys was comprised of interviewed households and their contacts with whom they exchanged pigs within the past year. Most contacts were not uniquely identifiable meaning that the personal, ‘egocentric’, networks of interviewees could not be linked. Nevertheless, important network characteristics were calculable for each smallholder type including: 1) the distributions of in- and out-degree centrality, 2) mixing patterns among smallholder types, and 3) the tendency for smallholders to trade with other smallholders within their village, district, province, or outside their province. We adopted a modified ‘configuration algorithm’, also referred to as a ‘matching algorithm’ (Bollobás, 1980; Britton et al., 2006), to simulate a directed, non-weighted swine trade network consistent with these observed characteristics following similar methods to Ferdousi (Ferdousi et al., 2019). The algorithm was coded in R (R Core Team, 2020), based on pseudocode in Ferdousi et al. (2019), to generate a direct contact network for each year of the simulation using following steps:

1. Assign directed in-and out-degree values (i.e. ‘stubs’) to each node by randomly sampling from the observed distributions of in- and out-degree for each actor.
2. Connect out-stubs to in-stubs of different nodes (respectively, senders and recipients) based on the mixing matrix between farm types and the distribution of locations connecting smallholders. If no appropriate match is found, expand the search to the next largest administrative unit (i.e. from village, to district, to province, to outside of province).

The indirect (fomites) network was simply the inverse of the direct (pig trade) network. Model fit was assessed by visual comparison of the three types of observed and simulated network characteristics described above.

*Spatial network generation*

The spatial contact network was generated by assigning undirected edges between nodes within a Euclidean distance of 1km from one another.

*Network edge dynamics*

Use of the simulated 1-year swine trade network in each 1-week time step would have provided a more connected network than the contagion could actually experience (Craft, 2015; Cross et al., 2005; Keeling and Eames, 2005). The 1-year network was therefore converted to a series of 1-week networks, considered appropriate to the minimum duration of herd-level persistence. Since the repeatability of edges (i.e. the frequency of trades between two smallholders) in the 1-year network was unknown, we assumed that all edges repeated in each 6-month production cycle. To achieve this, each edge was randomly allocated to a single week in a 6-month period. At each time step in the simulation, a 1-week trading network was then randomly selected without replacement. Meanwhile, the spatial network is fixed and so remained constant in every time step.

*Between-herd modelling transmission parameters*

Each smallholder, with its defined farm type and size, was assigned a fixed duration of IAV persistence and immunity based on the within-herd modelling results for smallholders with corresponding characteristics (Figure S3 and S4). Fattening farms were instead assigned a fixed duration of persistence and immunity (Canini et al., 2020). For each type of contact (direct, indirect, spatial), a range of transmission probabilities were explored (Table S1).

**References**

Allerson, M.W., Cardona, C.J., Torremorell, M., 2013. Indirect Transmission of Influenza A Virus between Pig Populations under Two Different Biosecurity Settings. PLOS ONE 8, e67293. https://doi.org/10.1371/journal.pone.0067293

Bollobás, B., 1980. A Probabilistic Proof of an Asymptotic Formula for the Number of Labelled Regular Graphs. Eur. J. Comb. 1, 311–316. https://doi.org/10.1016/S0195-6698(80)80030-8

Britton, T., Deijfen, M., Martin-Löf, A., 2006. Generating Simple Random Graphs with Prescribed Degree Distribution. J. Stat. Phys. 124, 1377–1397. https://doi.org/10.1007/s10955-006-9168-x

Canini, L., Holzer, B., Morgan, S., Hemmink, J.D., Clark, B., Consortium, sLoLa D., Woolhouse, M.E.J., Tchilian, E., Charleston, B., 2020. Timelines of infection and transmission dynamics of H1N1pdm09 in swine. PLOS Pathog. 16, e1008628. https://doi.org/10.1371/journal.ppat.1008628

Corzo, C.A., Culhane, M., Dee, S., Morrison, R.B., Torremorell, M., 2013. Airborne Detection and Quantification of Swine Influenza A Virus in Air Samples Collected Inside, Outside and Downwind from Swine Barns. PLOS ONE 8, e71444. https://doi.org/10.1371/journal.pone.0071444

Craft, M.E., 2015. Infectious disease transmission and contact networks in wildlife and livestock. Philos. Trans. R. Soc. Lond. B Biol. Sci. 370, 20140107. https://doi.org/10.1098/rstb.2014.0107

Cross, P.C., Lloyd-Smith, J.O., Johnson, P.L.F., Getz, W.M., 2005. Duelling timescales of host movement and disease recovery determine invasion of disease in structured populations. Ecol. Lett. 8, 587–595. https://doi.org/10.1111/j.1461-0248.2005.00760.x

Desrosiers, R., 2021. Survival and transmission of swine influenza A virus within and between farms. J. Swine Health Prod. 29, 133–138.

Desrosiers, R., Boutin, R., Broes, A., 2004. Persistence of antibodies after natural infection with swine influenza virus and epidemiology of the infection in a herd previously considered influenza-negative. J. Swine Health Prod. 12, 78–81.

Ferdousi, T., Moon, S.A., Self, A., Scoglio, C., 2019. Generation of swine movement network and analysis of efficient mitigation strategies for African swine fever virus. PLOS ONE 14, e0225785. https://doi.org/10.1371/journal.pone.0225785

Garrido-Mantilla, J., Sanhueza, J., Alvarez, J., Culhane, M.R., Davies, P., Allerson, M.W., Torremorell, M., 2021. Impact of nurse sows on influenza A virus transmission in pigs under field conditions. Prev. Vet. Med. 188, 105257. https://doi.org/10.1016/j.prevetmed.2021.105257

Gilbert, M., Nicolas, G., Cinardi, G., Van Boeckel, T.P., Vanwambeke, S.O., Wint, G.R.W., Robinson, T.P., 2018. Global distribution data for cattle, buffaloes, horses, sheep, goats, pigs, chickens and ducks in 2010. Sci. Data 5, 180227. https://doi.org/10.1038/sdata.2018.227

Hu, Z., Tian, X., Lai, R., Ji, C., Li, X., 2023. Airborne transmission of common swine viruses. Porc. Health Manag. 9, 50. https://doi.org/10.1186/s40813-023-00346-6

Keeling, M.J., Eames, K.T.D., 2005. Networks and epidemic models. J. R. Soc. Interface 2, 295–307. https://doi.org/10.1098/rsif.2005.0051

Poljak, Z., Friendship, R.M., Carman, S., McNab, W.B., Dewey, C.E., 2008. Investigation of exposure to swine influenza viruses in Ontario (Canada) finisher herds in 2004 and 2005. Prev. Vet. Med. 83, 24–40. https://doi.org/10.1016/j.prevetmed.2007.05.025

Thompson, K.-A., Bennett, A.M., 2017. Persistence of influenza on surfaces. J. Hosp. Infect. 95, 194–199. https://doi.org/10.1016/j.jhin.2016.12.003

Torremorell, M., Allerson, M., Corzo, C., Diaz, A., Gramer, M., 2012. Transmission of Influenza A Virus in Pigs. Transbound. Emerg. Dis. 59, 68–84. https://doi.org/10.1111/j.1865-1682.2011.01300.x

Zeller, M.A., Ma, J., Wong, F.Y., Tum, S., Hidano, A., Holt, H., Chhay, T., Sorn, S., Koeut, D., Seng, B., Chao, S., Ng, G.G.K., Yan, Z., Chou, M., Rudge, J.W., Smith, G.J.D., Su, Y.C.F., 2023. The genomic landscape of swine influenza A viruses in Southeast Asia. Proc. Natl. Acad. Sci. 120, e2301926120. https://doi.org/10.1073/pnas.2301926120

**Table S1. Transmission parameters for between-herd IAV modelling estimated by within-herd model**

| **Parameter** | **Value(s)**  **median (lower quartile, upper quartile; max)** | **Justification** |
| --- | --- | --- |
| **Duration of persistence (days)** |  |  |
| breeding-No boar | 10 (6, 22; 103) | Within herd simulations |
| breeding-Boar | 10 (6, 27; 103) | Within herd simulations |
| BSP | 14 (6, 6; 61) | Within herd simulations |
| fattening-No boar | 6 (6, 6; 6) | (Canini et al., 2020) |
| FtoF-No boar | 10 (6, 22; 103) | Within herd simulations |
| FtoF-Boar | 10 (6, 27; 103) | Within herd simulations |
| **Duration of immunity (days)** |  |  |
| breeding-No boar | 196 (183, 208; 350) | Within herd simulations |
| breeding-Boar | 197 (184, 210; 881) | Within herd simulations |
| BSP | 183 (170, 197; 428) | Within herd simulations |
| fattening-No boar | 180 (180, 180; 180) | 1 production cycle |
| FtoF-No boar | 196 (183, 208; 350) | Within herd simulations |
| FtoF-Boar | 197 (184, 210; 881) | Within herd simulations |
| **Transmission probability** |  |  |
| Direct: high  Direct: low | 1  0.8 | Dorjee et al. (2016)  Assumed |
| Indirect: high  Indirect: medium  Indirect: low | 0.2  0.1  0.01 | Assumed  Assumed  Dorjee et al. (2016) |
| Spatial: high  Spatial: medium  Spatial: low | 0.2  0.1  0.01 | Assumed  Assumed  Assumed |

**Table S2. Demographics of smallholders in Oudomxay and Luang Namtha province**

|  | Province | | | |  |
| --- | --- | --- | --- | --- | --- |
| Variable | Oudomxay (n = 117) |  | Luang Namtha (n = 65) |  |  |
| **Gender** |  |  |  |  |  |
| Male | 58 | (49.6%) | 30 | (46.2%) |  |
|  |  |  |  |  |  |
| **Age^1^** | 45 | (19-81) | 48 | (20-79) |  |
|  |  |  |  |  |  |
| **Ethnicity** |  |  |  |  |  |
| Khmu | 38 | (32.5%) | 19 | (29.2%) |  |
| Hmong | 18 | (15.4%) | 0 | (0%) |  |
| Lao-tai | 1 | (0.9%) | 23 | (35.4%) |  |
| Lao | 14 | (12.0%) | 11 | (16.9%) |  |
| Other | 46 | (39.3%) | 12 | (18.5%) |  |
|  |  |  |  |  |  |
| **Education** | |  |  |  |  |
| Primary or lower | 74 | (63.2%) | 39 | (60%) |  |
| Secondary | 34 | (29.1%) | 20 | (30.8%) |  |
| College, university or higher | 9 | (7.7%) | 6 | (9.2%) |  |
|  |  |  |  |  |  |
| **Operation years^1^** | 3.5 | (0.2 - 25) | 3 | (0.1 - 40) |  |

^1^ Median and range are shown

**Table S3. Distribution of litter sizes across smallholder type in northern Laos**

|  | Litter size | | |
| --- | --- | --- | --- |
| Typology | Median | Q1, Q3 | Range |
| Boar service provider (n = 12) | 8 | 7, 10 | (5 - 12) |
| Breeding with boar (n = 17) | 9 | 8,10 | (5 - 13) |
| Breeding without boar (n = 16) | 6 | 3.75, 10 | (1 - 12) |
| Fattening (n = 68) | NA |  |  |
| Farrow-to-finish with boar (n = 24) | 9.5 | 8, 10 | (4 - 15) |
| Farrow-to-finish without boar (n = 45) | 7 | 5, 10 | (0 - 15) |

**Table S4. Distribution of the mode of replacing sows across smallholder type in northern Laos**

| Type | Use own gilt | Introduce from other villages | Introduce from same village | Use own & introduce from other village | Use own & introduce from commercial  farm |
| --- | --- | --- | --- | --- | --- |
| Boar service provider (n = 9) | 9 (100%) | 0 | 0 | 0 | 0 |
| Breeding with boar (n = 17) | 15 (88.2%) | 2 (11.8%) | 0 | 0 | 0 |
| Breeding without boar (n = 16) | 14 (87.5%) | 0 | 2 (12.5%) | 0 | 0 |
| Farrow-to-finish with boar (n = 24) | 20 (83.3%) | 1 (4.2%) | 1 (4.2%) | 1 (4.2%) | 1 (4.2%) |
| Farrow-to-finish without boar (n = 45) | 34 (75.6%) | 3 (6.7%) | 8 (17.8%) | 0 | 0 |

**Table S5. Distribution of the mode of boar usage across smallholder type in northern Laos**

| Type | Own boar | Hire boar |
| --- | --- | --- |
| Boar service provider (n = 9) | 8 (88.9%) | 1 (11.1%) |
| Breeding with boar (n = 17) | 17 (100%) | 0 (0%) |
| Breeding without boar (n = 16) | 3 (18.8%) | 13 (81.2%) |
| Farrow-to-finish with boar (n = 24) | 22 (91.7%) | 2 (8.3%) |
| Farrow-to-finish without boar (n = 45) | 7 (15.6%) | 38 (84.4%) |

**Table S6. Results of multivariable fractional polynomial regressions for the persistence and immune durations across smallholder type**

| **Smallholder type** | **Persistence** | |  | **Immune duration** | |
| --- | --- | --- | --- | --- | --- |
| Functional form of variables | Coefficient (95%CI) | p value |  | Coefficient (95%CI) | p value |
| Boar service provider |  |  |  |  |  |
| log(Sow/10) | 4.04 (3.64 - 4.45) | <0.01 |  | -37.1 (-38.2 - -36.0) | <0.01 |
| log(Sow/10)^2 | 0.49 (0.31 - 0.66) | <0.01 |  |  |  |
| Boar | 0.89 (0.72 - 1.05) | <0.01 |  | 0.48 (-1.22 - 2.18) | 0.58 |
|  |  |  |  |  |  |
| Breeding with fattening |  |  |  |  |  |
| (Sow/10)^-1 | 1.44 (1.24 - 1.65) | <0.01 |  | 33.3 (31.0 - 35.7) | <0.01 |
| (Sow/10)^-0.5 | -9.66 (-10.4 - -8.94) | <0.01 |  |  |  |
| (Sow/10)^-1 * log(Sow/10) |  |  |  | 9.02 (8.10 - 9.95) | <0.01 |
| Boar | 0.57 (0.32 - 0.81) | <0.01 |  | -2.91 (-4.42 - -1.39) | <0.01 |
|  |  |  |  |  |  |
| Breeding without fattening |  |  |  |  |  |
| (Sow/10)^-2 | 0.06 (0.05 - 0.07) | <0.01 |  |  |  |
| (Sow/10)^-0.5 | -6.03 (-6.38 - -5.68) | <0.01 |  |  |  |
| log(Sow/10) |  |  |  | -65.3 (-68.0 - -62.6) | <0.01 |
| (Sow/10)^0.5 |  |  |  | 83.5 (77.0 - 90.0) | <0.01 |
| Boar | 0.74 (0.54 - 0.95) | <0.01 |  | -2.06 (-3.70 - -0.43) | 0.01 |

**Table S7. Comparison of distribution of locations in which trade partners were located as observed in the empirical data, and in the simulated swine trade network.**

|  | **Observed** | **Simulated** |
| --- | --- | --- |
| **Location of trade partner** | **(Proportion)** | |
| **Same village** | 0.81 | 0.79 |
| **Same district** | 0.14 | 0.15 |
| **Same province** | 0.02 | 0.02 |
| **Different province** | 0.03 | 0.04 |

**Table S8. Network statistics of simulated networks and their equivalent Erdős–Rényi random graphs with the same number of nodes and edges.** WCC=weakly connected components; SCC=strongly connected components; cells are shaded for the spatial network which was undirected.

| **Network** | **Density** | **No. edges** | **Prop. Iso.** | **No. WCC** | **Size of largest WCC** | **No. SCC** | **Size of largest SCC** | **Average geodesic** | **Global clustering coefficient** |
| --- | --- | --- | --- | --- | --- | --- | --- | --- | --- |
| Pig trade: 1 year | 3.27E-04 | 5237 | 0.31 | 120 | 2449 | 82 | 697 | 11.15 | 0.02 |
| Erdős–Rényi | 3.27E-04 | 5237 | 0.07 | 41 | 3604 | 3 | 725 | 21.80 | 0.00 |
| Pig trade: 1 week | 1.29E-05 | 206 | 0.91 | 154 | 6 | 2 | 2 | 1.00 | 0.00 |
| Erdős–Rényi | 1.29E-05 | 206 | 0.90 | 186 | 5 | 0 | 1 | 1.20 | 0.00 |
| Spatial: 1 year | 1.09E-03 | 17400 | 0.02 | 153 | 1130 |  |  | 32.02 | 0.59 |
| Erdős–Rényi | 1.09E-03 | 17400 | 0.00 | 1 | 4000 |  |  | 5.79 | 0.00 |

***
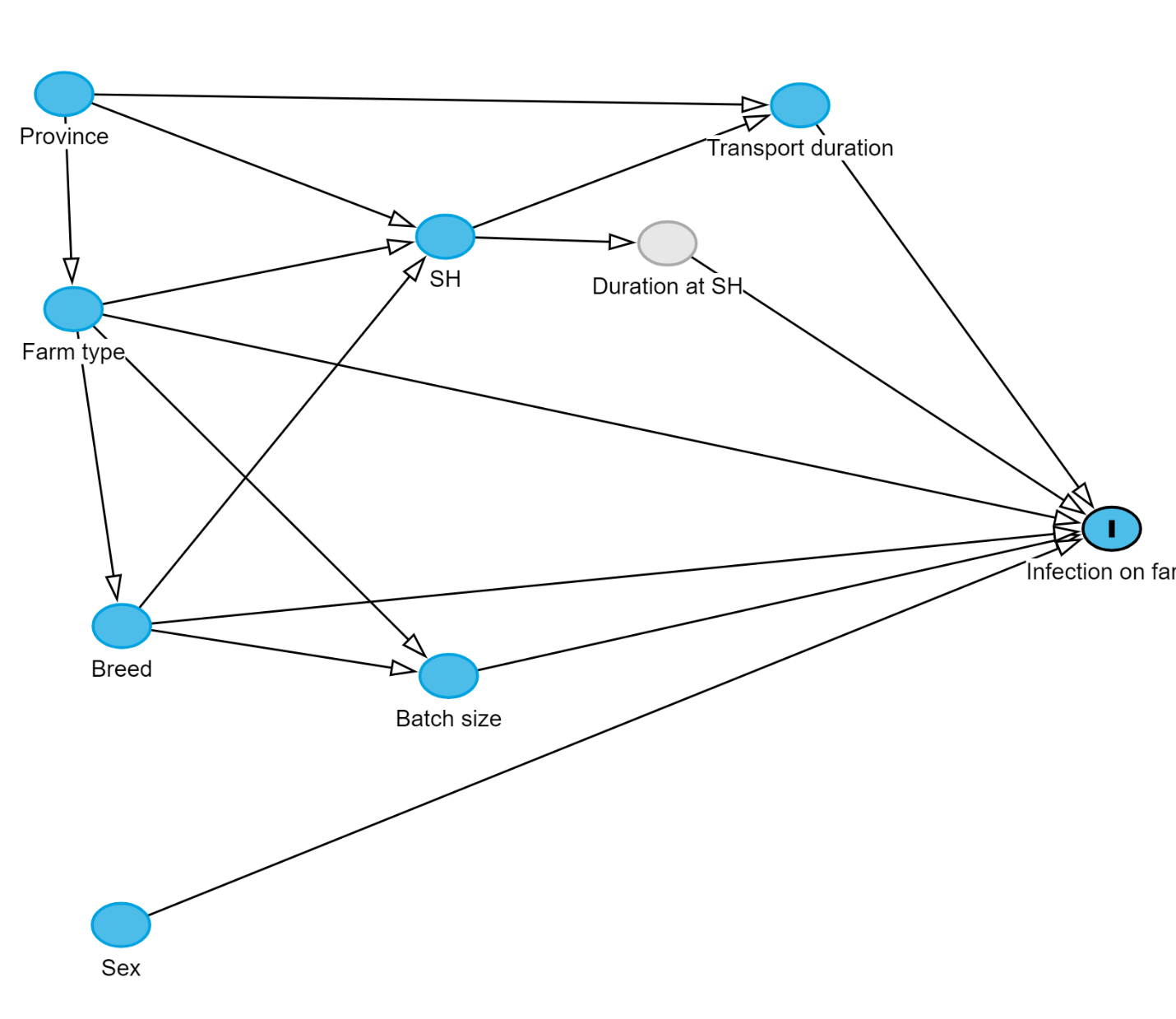
***

**Figure S1. Directed acyclic graph for the assumed causal relationship between explanatory variables and the outcome (infection on farm), which was approximated by the ELISA status.**


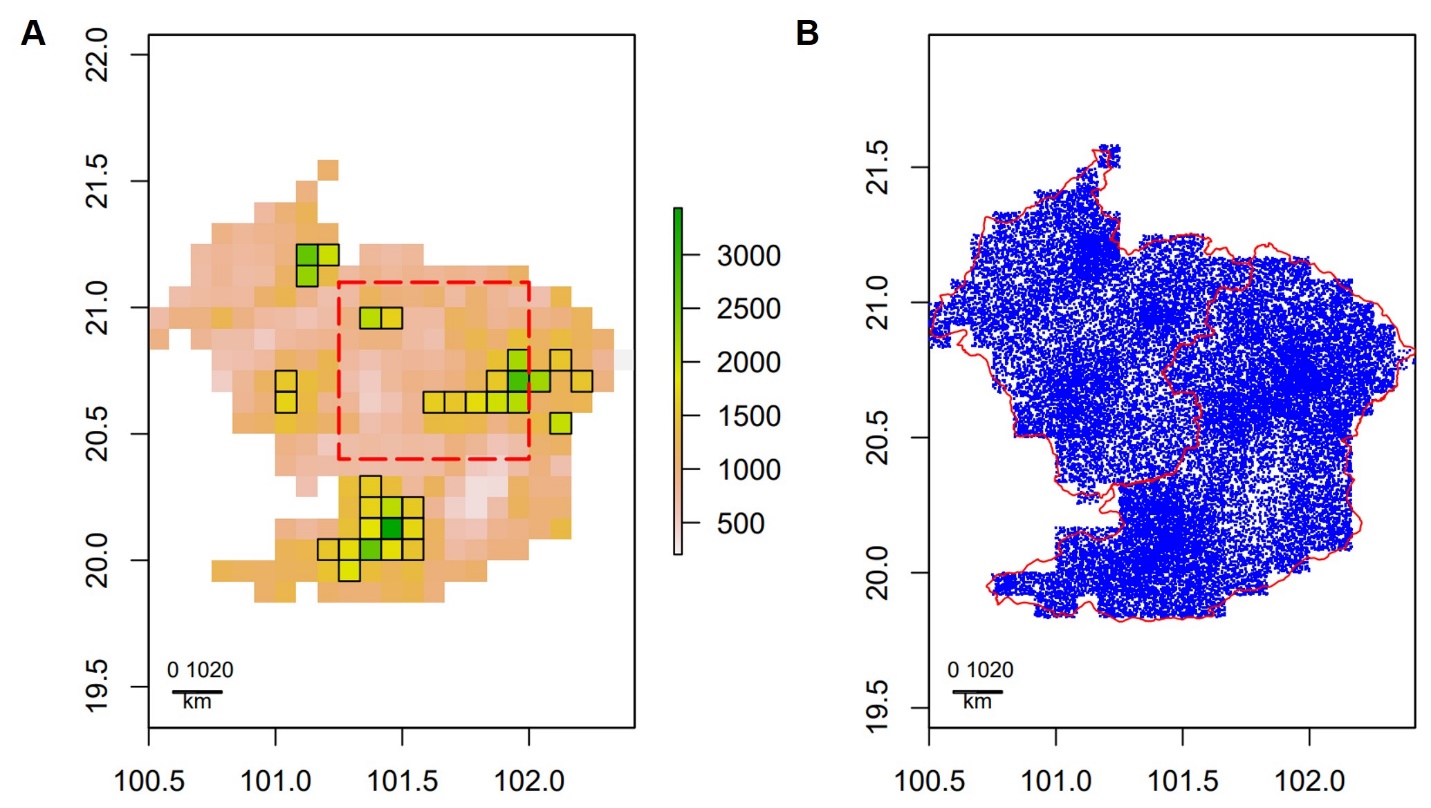


**
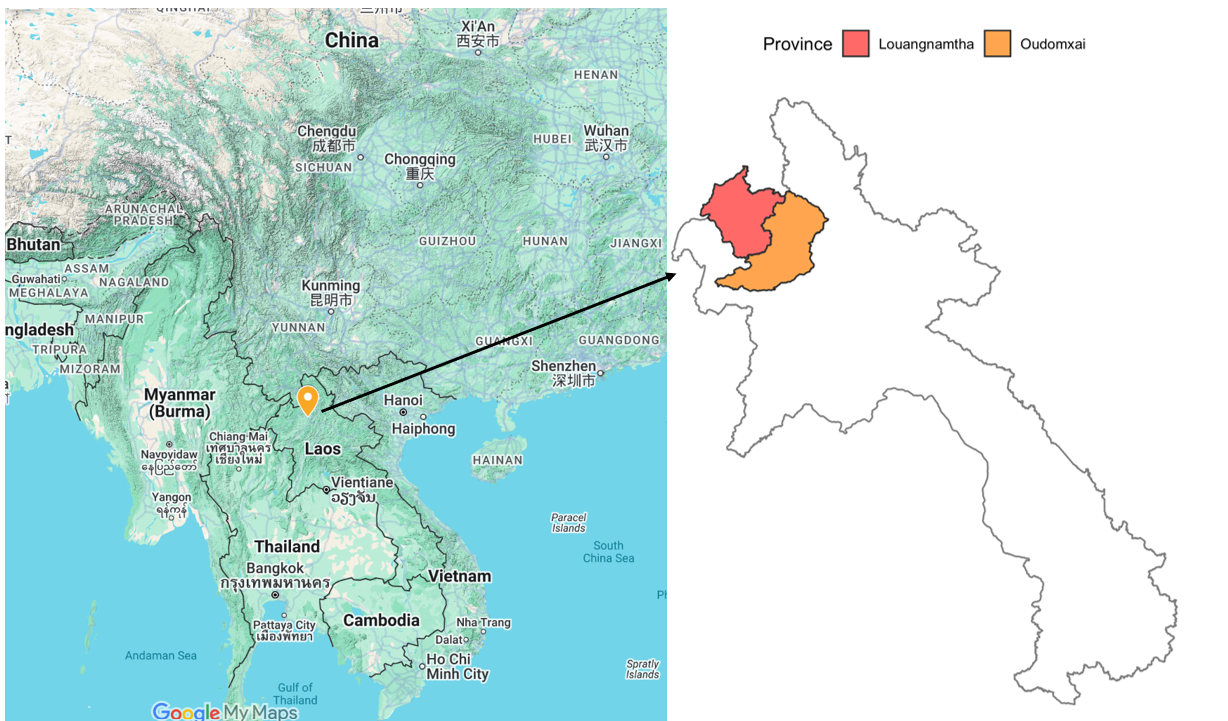
 C**

**Figure S2. Map of the study site.** (A) Pig densities within the study area based on Gridded Livestock of the World data. Grid cells defined as high pig density areas are marked with a black border and the selected simulation study area is shown as a red dashed box. (B) Distribution of simulated smallholder nodes. (C) Map of the study location (Oudomxay in orange and Luang Namtha in red) in relation to bordering countries


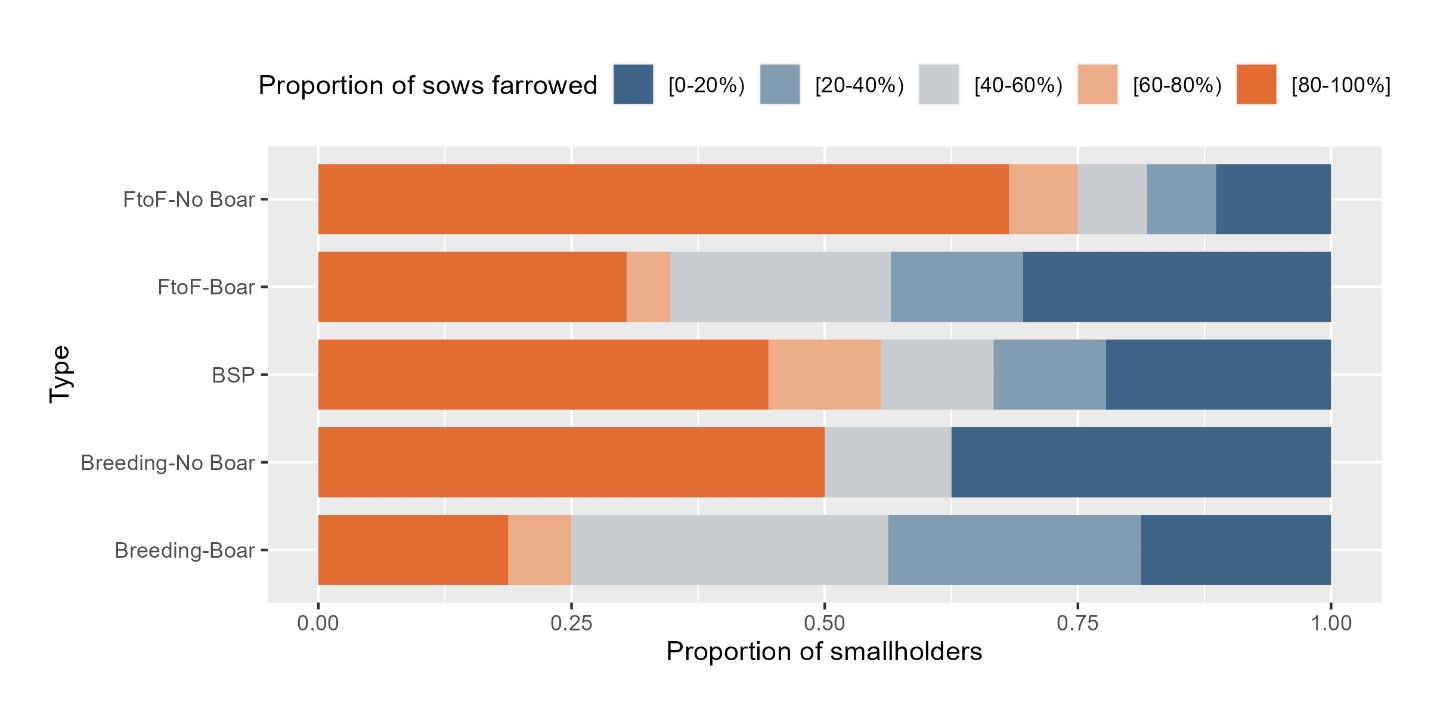


**Figure S3. Distributions of the proportion of sows that farrowed in the past 3 months stratified by smallholder type in northern Laos**. Each colour represents the category of the proportion of sows that farrowed in the past 3 months where [0-20%] indicates that less than 20% of sows in herd farrowed in the last 3 months etc.


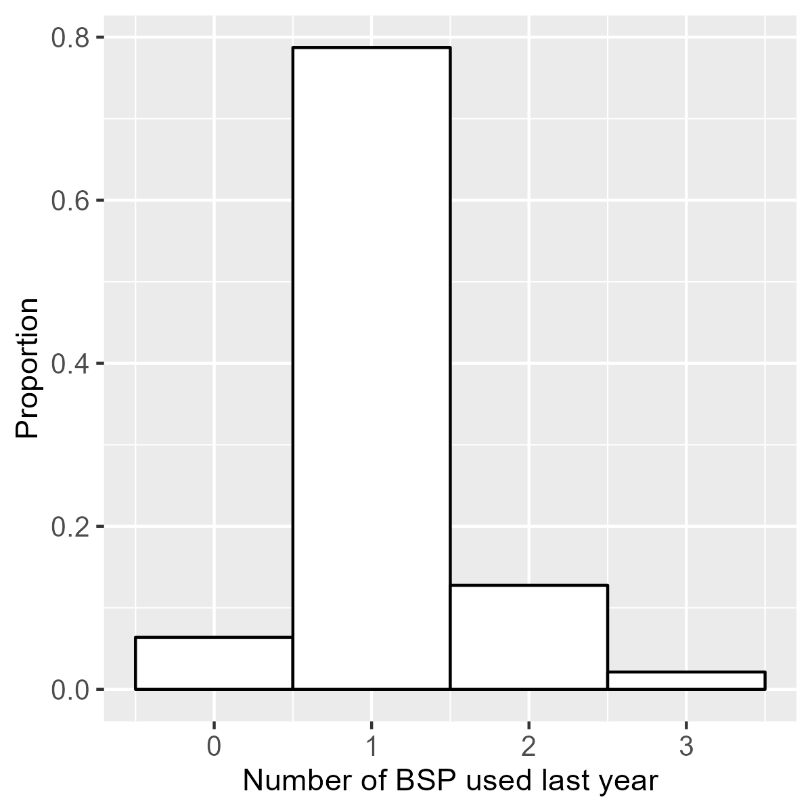


**Figure S4. Number of different boar service providers (BSPs) used by smallholders, who hired boars, in the past 1 year in northern Laos**

**
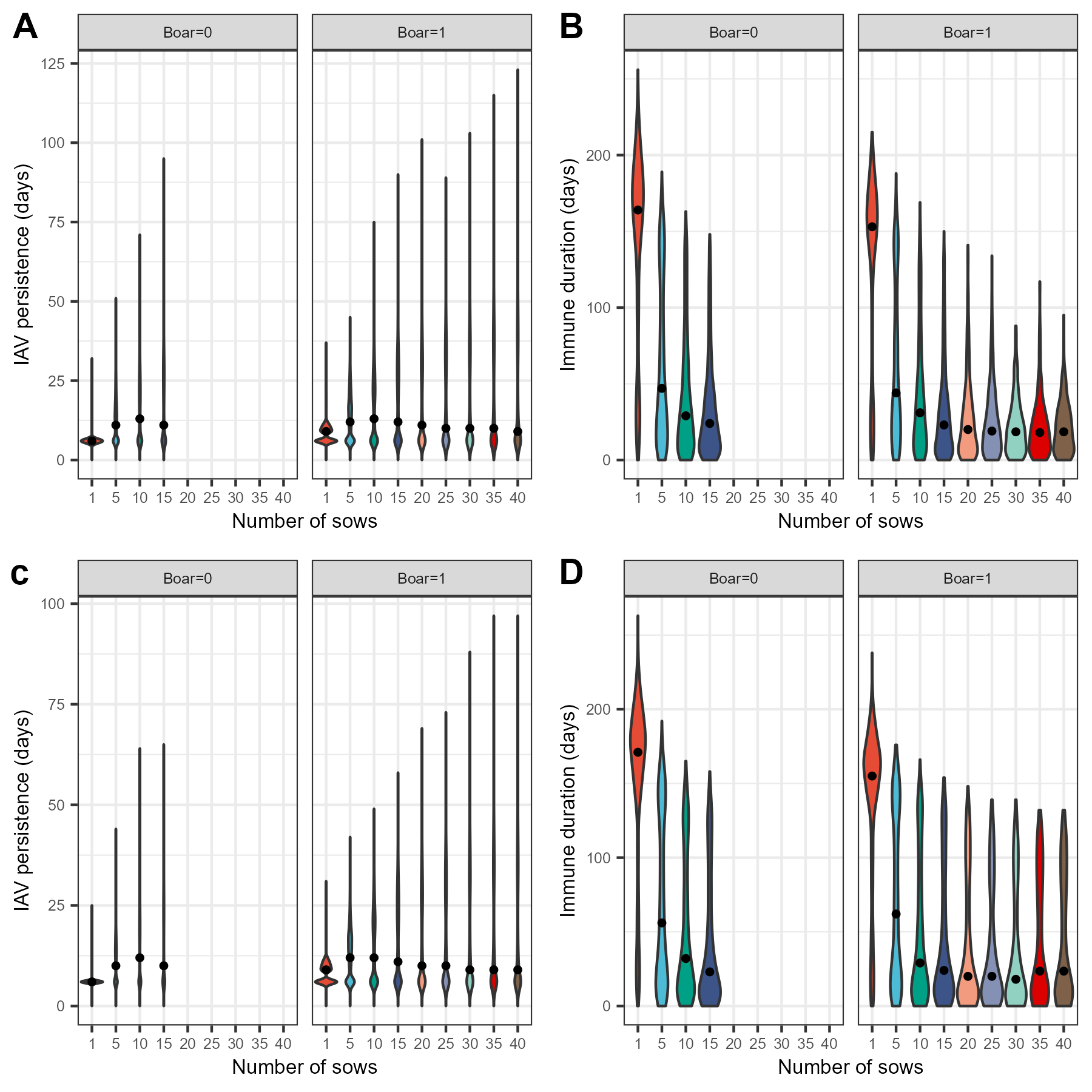
**

**Figure S5. Distributions of the simulated persistence and immune duration across smallholder type.** (A) IAV persistence and (B) immune duration of farrow-to-finish smallholders and (C) IAV persistence and (D) immune duration of breeding smallholders, stratified by the number of sows and boars


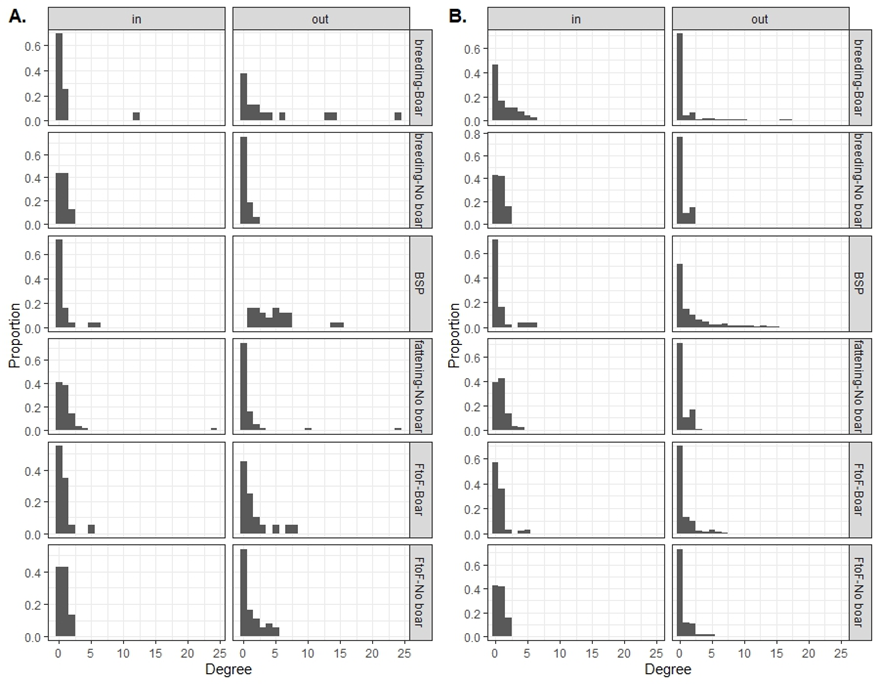


**Figure S6. Comparison of distributions of in- and out-degree by smallholder type. (A) observed pig trade data and (B) simulated pig trade network.**


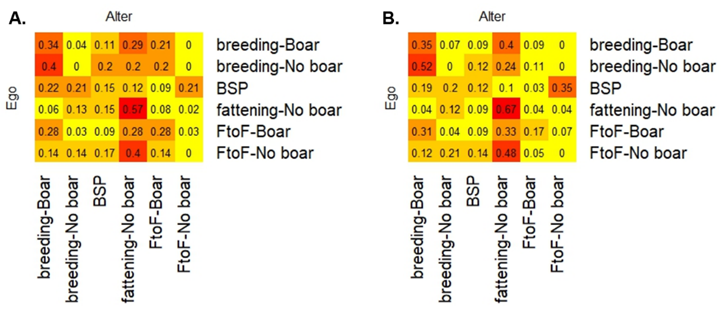


**Figure S7. Comparison of mixing matrices by smallholder type. (A) observed pig trade data and (B) simulated pig trade network. Cells represent, row-wise for each ego, the proportion of their contacts that belonged to each type.**


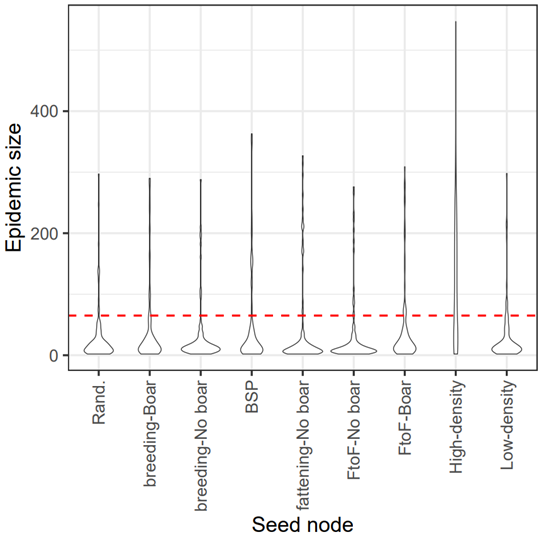


**Figure S8. Violin plot of the distribution of epidemic sizes following seeding in different smallholder types and pig density regions. (Rand = total random seeding). The selected cut-off of 65 infected nodes is shown (dotted red line)**


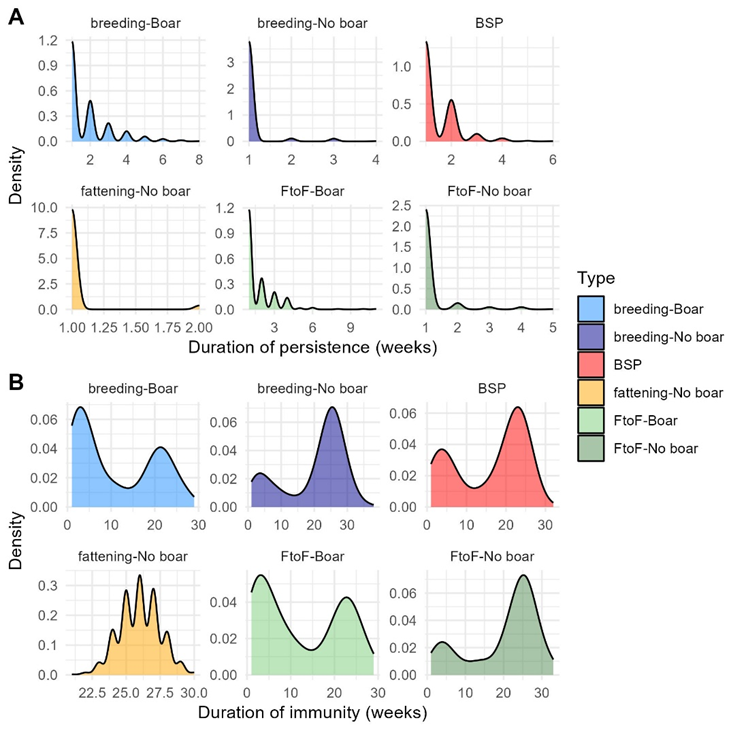


**Figure S9. Distributions of duration of node-level persistence (A) and immunity (B) of the simulated population of nodes.**


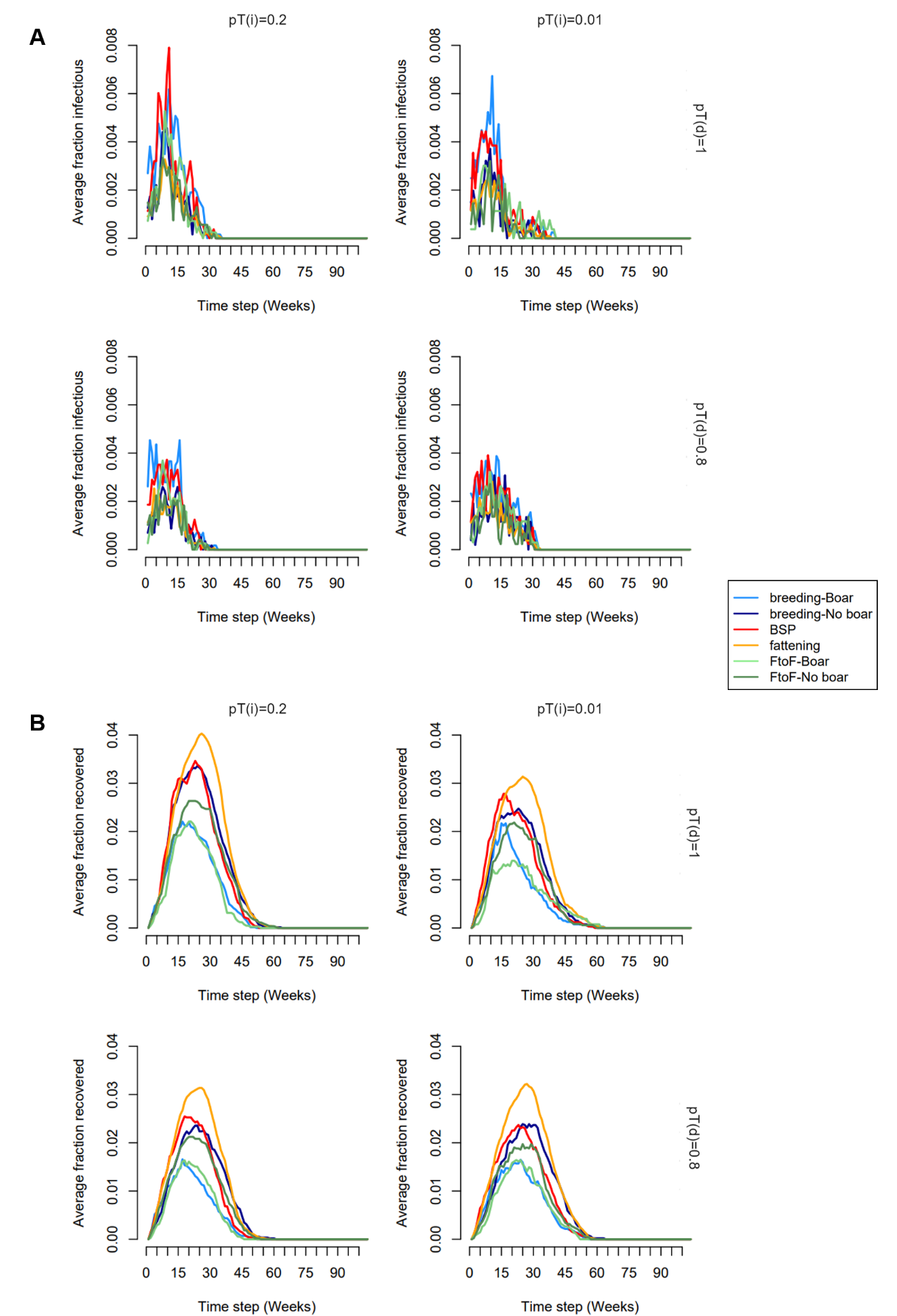


**Figure S10. Infectious disease dynamics by actor. The mean proportion of infectious (A) and recovered (B) nodes over the course of the simulation, shown here for all transmissibility scenarios following random actor seeding.**


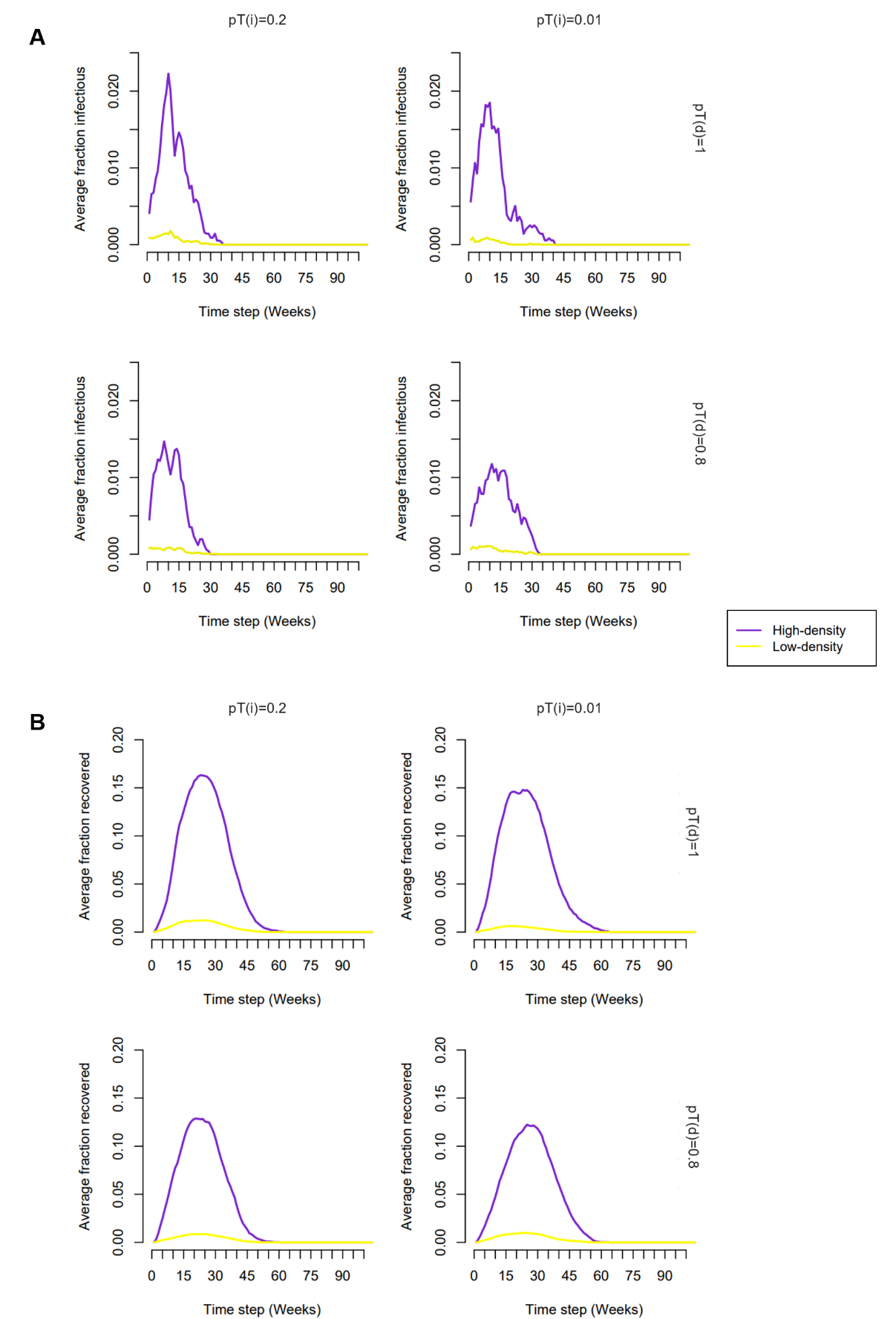


**Figure S11. Infectious disease dynamics by region. The mean proportion of infectious (A) and recovered (B) nodes over the course of the simulation, shown here for all transmissibility scenarios following random actor seeding.**


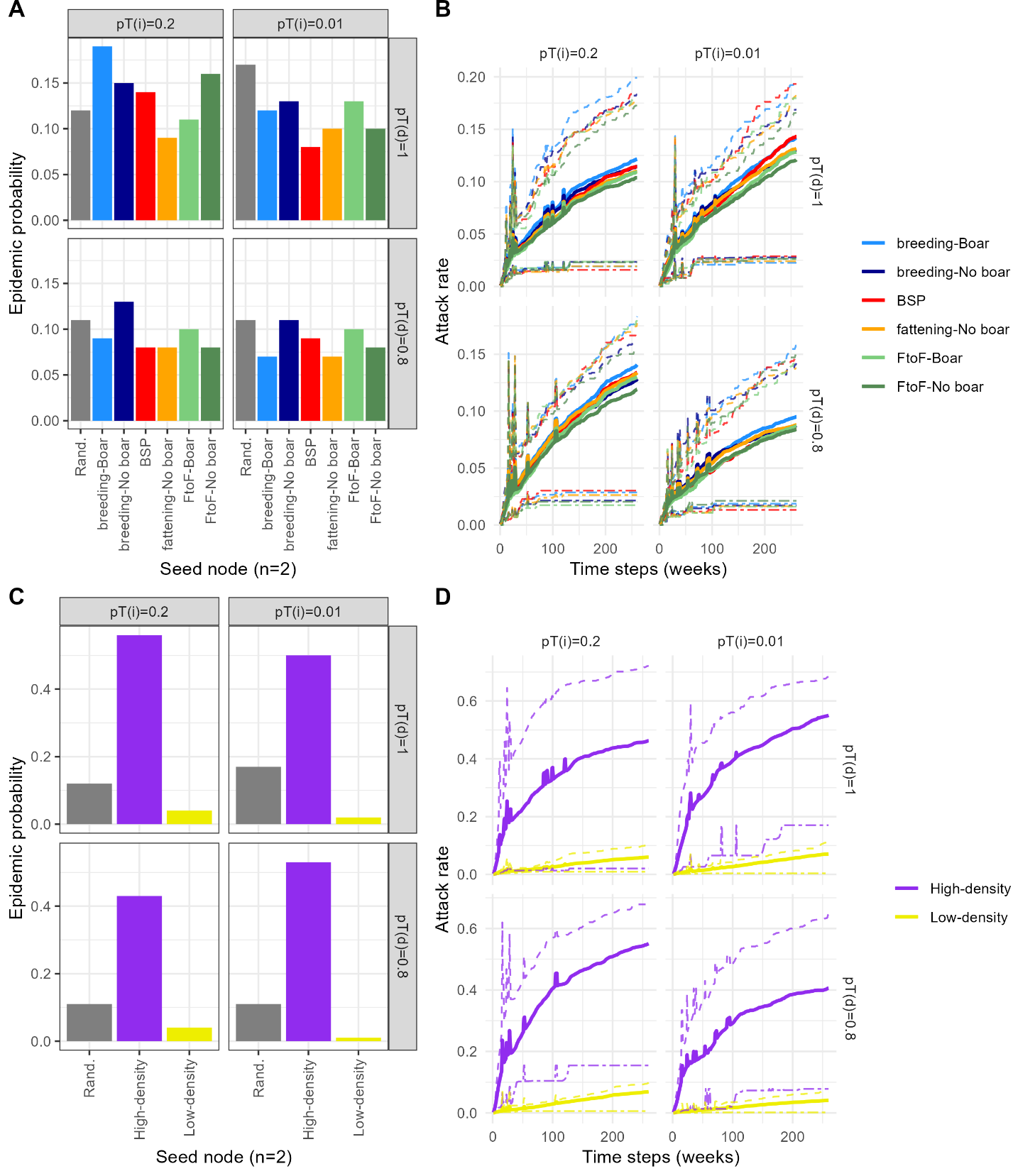
**Figure S12. Scenarios of persistently infected large commercial farms. Epidemic probability after seeding in defined smallholder types (A) and in different pig density regions (C) (Rand = total random seeding) and cumulative epidemic attack rate by smallholder type (B) and pig density region (D) following total random seeding. Solid lines show median cumulative epidemic attack rate; dashed lines show inter-95-percentiles across 100 iterations.**
