# Supplementary material for: Epidemiology and Risk Modelling of Influenza A Virus Within and Between Pig Herds in Northern Lao PDR
Source: Transbound Emerg Dis. 2026 Jan 23;2026:2407533. doi: 10.1155/tbed/2407533 (PMC12829471; doi:10.1155/tbed/2407533)
Supplement: Supplementary file 1 — Supporting Information Table S1. Transmission parameters for between‐herd influenza A virus (IAV) modelling estimated by within‐herd model. Table S2. Demographics of smallholders in Oudomxay and Luang Namtha province. Table S3. Distribution of litter sizes across smallholder type in northern Laos. Table S4. Distribution of the mode of replacing sows across smallholder type in northern Laos. Table S5. Distribution of the mode of boar usage across smallholder type in northern Laos. Table S6. Results of multivariable fractional polynomial regressions for the persistence and immune durations across smallholder type. Table S7. Comparison of distribution of locations in which trade partners were located as observed in the empirical data and in the simulated swine trade network. Table S8. Network statistics of simulated networks and their equivalent Erdős–Rényi random graphs with the same number of nodes and edges. Figure S1. Directed acyclic graph for the assumed causal relationship between explanatory variables and the outcome (infection on farm), which was approximated by the ELISA status. Figure S2. Map of the study site. Figure S3. Distributions of the proportion of sows that farrowed in the past 3 months stratified by smallholder type in northern Laos. Figure S4. Number of different boar service providers (BSPs) used by smallholders, who hired boars, in the past 1 year in northern Laos. Figure S5. Distributions of the simulated persistence and immune duration across smallholder type. Figure S6. Comparison of distributions of in‐ and out‐degree by smallholder type. Figure S7. Comparison of mixing matrices by smallholder type. Figure S8. Violin plot of the distribution of epidemic sizes following seeding in different smallholder types and pig density regions. Figure S9. Distributions of duration of node‐level persistence (A) and immunity (B) of the simulated population of nodes. Figure S10. Infectious disease dynamics by actor. Figure S11. Infectious disease dynamics b [file TBED-2026-2407533-s001.zip › RISNIP_draft_Supplement_modelling_v4_Apr2025.docx]

Supplementary Materials

# Epidemiology and risk modelling of Influenza A virus within and between pig herds in northern Lao PDR

Network characteristics

A large weakly connected components (WCC) of 2449 nodes was present – representing the maximum extent of disease spread on a static network of this structure. Strongly connected components (SCC) are subgraphs in which all nodes are reachable from one another while taking into consideration edge directionality. More SCCs were present than in the equivalent random graph. The simulated spatial network was, as expected, lattice-like in structure with high clustering and long average geodesics relative to an equivalent random graph.

**Table S1. Transmission parameters for between-herd IAV modelling estimated by within-herd model**

| **Parameter** | **Value(s)**  **median (lower quartile, upper quartile; max)** | **Justification** |
| --- | --- | --- |
| **Duration of persistence (days)** |  |  |
| breeding-No boar | 10 (6, 22; 103) | Within herd simulations |
| breeding-Boar | 10 (6, 27; 103) | Within herd simulations |
| BSP | 14 (6, 6; 61) | Within herd simulations |
| fattening-No boar | 6 (6, 6; 6) | (Canini et al., 2020) |
| FtoF-No boar | 10 (6, 22; 103) | Within herd simulations |
| FtoF-Boar | 10 (6, 27; 103) | Within herd simulations |
| **Duration of immunity (days)** |  |  |
| breeding-No boar | 196 (183, 208; 350) | Within herd simulations |
| breeding-Boar | 197 (184, 210; 881) | Within herd simulations |
| BSP | 183 (170, 197; 428) | Within herd simulations |
| fattening-No boar | 180 (180, 180; 180) | 1 production cycle |
| FtoF-No boar | 196 (183, 208; 350) | Within herd simulations |
| FtoF-Boar | 197 (184, 210; 881) | Within herd simulations |
| **Transmission probability** |  |  |
| Direct: high  Direct: low | 1  0.8 | Dorjee et al. (2016)  Assumed |
| Indirect: high  Indirect: medium  Indirect: low | 0.2  0.1  0.1  0.01 | Assumed  Assumed  Dorjee et al. (2016) |
| Spatial: high  Spatial: medium  Spatial: low | 0.2  0.1  0.01 | Assumed  Assumed  Assumed |

**Table S2. Demographics of smallholders in Oudomxay and Luang Namtha province**

|  | Province | | | |  |
| --- | --- | --- | --- | --- | --- |
| Variable | Oudomxay (n = 117) |  | Luang Namtha (n = 65) |  |  |
| **Gender** |  |  |  |  |  |
| Male | 58 | (49.6%) | 30 | (46.2%) |  |
|  |  |  |  |  |  |
| **Age^1^** | 45 | (19-81) | 48 | (20-79) |  |
|  |  |  |  |  |  |
| **Ethnicity** |  |  |  |  |  |
| Khmu | 38 | (32.5%) | 19 | (29.2%) |  |
| Hmong | 18 | (15.4%) | 0 | (0%) |  |
| Lao-tai | 1 | (0.9%) | 23 | (35.4%) |  |
| Lao | 14 | (12.0%) | 11 | (16.9%) |  |
| Other | 46 | (39.3%) | 12 | (18.5%) |  |
|  |  |  |  |  |  |
| **Education** | |  |  |  |  |
| Primary or lower | 74 | (63.2%) | 39 | (60%) |  |
| Secondary | 34 | (29.1%) | 20 | (30.8%) |  |
| College, university or higher | 9 | (7.7%) | 6 | (9.2%) |  |
|  |  |  |  |  |  |
| **Operation years^1^** | 3.5 | (0.2 - 25) | 3 | (0.1 - 40) |  |

^1^ Median and range are shown

**Table S3. Distribution of litter sizes across smallholder type in northern Laos**

|  | Litter size | | |
| --- | --- | --- | --- |
| Typology | Median | Q1, Q3 | Range |
| Boar service provider (n = 12) | 8 | 7, 10 | (5 - 12) |
| Breeding with boar (n = 17) | 9 | 8,10 | (5 - 13) |
| Breeding without boar (n = 16) | 6 | 3.75, 10 | (1 - 12) |
| Fattening (n = 68) | NA |  |  |
| Farrow-to-finish with boar (n = 24) | 9.5 | 8, 10 | (4 - 15) |
| Farrow-to-finish without boar (n = 45) | 7 | 5, 10 | (0 - 15) |

**Table S4. Distribution of the mode of replacing sows across smallholder type in northern Laos**

| Type | Use own gilt | Introduce from other villages | Introduce from same village | Use own & introduce from other village | Use own & introduce from commercial  farm |
| --- | --- | --- | --- | --- | --- |
| Boar service provider (n = 9) | 9 (100%) | 0 | 0 | 0 | 0 |
| Breeding with boar (n = 17) | 15 (88.2%) | 2 (11.8%) | 0 | 0 | 0 |
| Breeding without boar (n = 16) | 14 (87.5%) | 0 | 2 (12.5%) | 0 | 0 |
| Farrow-to-finish with boar (n = 24) | 20 (83.3%) | 1 (4.2%) | 1 (4.2%) | 1 (4.2%) | 1 (4.2%) |
| Farrow-to-finish without boar (n = 45) | 34 (75.6%) | 3 (6.7%) | 8 (17.8%) | 0 | 0 |

**Table S5. Distribution of the mode of boar usage across smallholder type in northern Laos**

| Type | Own boar | Hire boar |
| --- | --- | --- |
| Boar service provider (n = 9) | 8 (88.9%) | 1 (11.1%) |
| Breeding with boar (n = 17) | 17 (100%) | 0 (0%) |
| Breeding without boar (n = 16) | 3 (18.8%) | 13 (81.2%) |
| Farrow-to-finish with boar (n = 24) | 22 (91.7%) | 2 (8.3%) |
| Farrow-to-finish without boar (n = 45) | 7 (15.6%) | 38 (84.4%) |

**Table S6. Results of multivariable fractional polynomial regressions for the persistence and immune durations across smallholder type**

| **Smallholder type** | **Persistence** | |  | **Immune duration** | |
| --- | --- | --- | --- | --- | --- |
| Functional form of variables | Coefficient (95%CI) | p value |  | Coefficient (95%CI) | p value |
| Boar service provider |  |  |  |  |  |
| log(Sow/10) | 4.04 (3.64 - 4.45) | <0.01 |  | -37.1 (-38.2 - -36.0) | <0.01 |
| log(Sow/10)^2 | 0.49 (0.31 - 0.66) | <0.01 |  |  |  |
| Boar | 0.89 (0.72 - 1.05) | <0.01 |  | 0.48 (-1.22 - 2.18) | 0.58 |
|  |  |  |  |  |  |
| Breeding with fattening |  |  |  |  |  |
| (Sow/10)^-1 | 1.44 (1.24 - 1.65) | <0.01 |  | 33.3 (31.0 - 35.7) | <0.01 |
| (Sow/10)^-0.5 | -9.66 (-10.4 - -8.94) | <0.01 |  |  |  |
| (Sow/10)^-1 * log(Sow/10) |  |  |  | 9.02 (8.10 - 9.95) | <0.01 |
| Boar | 0.57 (0.32 - 0.81) | <0.01 |  | -2.91 (-4.42 - -1.39) | <0.01 |
|  |  |  |  |  |  |
| Breeding without fattening |  |  |  |  |  |
| (Sow/10)^-2 | 0.06 (0.05 - 0.07) | <0.01 |  |  |  |
| (Sow/10)^-0.5 | -6.03 (-6.38 - -5.68) | <0.01 |  |  |  |
| log(Sow/10) |  |  |  | -65.3 (-68.0 - -62.6) | <0.01 |
| (Sow/10)^0.5 |  |  |  | 83.5 (77.0 - 90.0) | <0.01 |
| Boar | 0.74 (0.54 - 0.95) | <0.01 |  | -2.06 (-3.70 - -0.43) | 0.01 |

**Table S7. Comparison of distribution of locations in which trade partners were located as observed in the empirical data, and in the simulated swine trade network.**

|  | **Observed** | **Simulated** |
| --- | --- | --- |
| **Location of trade partner** | **(Proportion)** | |
| **Same village** | 0.81 | 0.79 |
| **Same district** | 0.14 | 0.15 |
| **Same province** | 0.02 | 0.02 |
| **Different province** | 0.03 | 0.04 |

**Table S8. Network statistics of simulated networks and their equivalent Erdős–Rényi random graphs with the same number of nodes and edges.** WCC=weakly connected components; SCC=strongly connected components; cells are shaded for the spatial network which was undirected.

| **Network** | **Density** | **No. edges** | **Prop. Iso.** | **No. WCC** | **Size of largest WCC** | **No. SCC** | **Size of largest SCC** | **Average geodesic** | **Global clustering coefficient** |
| --- | --- | --- | --- | --- | --- | --- | --- | --- | --- |
| Pig trade: 1 year | 3.27E-04 | 5237 | 0.31 | 120 | 2449 | 82 | 697 | 11.15 | 0.02 |
| Erdős–Rényi | 3.27E-04 | 5237 | 0.07 | 41 | 3604 | 3 | 725 | 21.80 | 0.00 |
| Pig trade: 1 week | 1.29E-05 | 206 | 0.91 | 154 | 6 | 2 | 2 | 1.00 | 0.00 |
| Erdős–Rényi | 1.29E-05 | 206 | 0.90 | 186 | 5 | 0 | 1 | 1.20 | 0.00 |
| Spatial: 1 year | 1.09E-03 | 17400 | 0.02 | 153 | 1130 |  |  | 32.02 | 0.59 |
| Erdős–Rényi | 1.09E-03 | 17400 | 0.00 | 1 | 4000 |  |  | 5.79 | 0.00 |

***
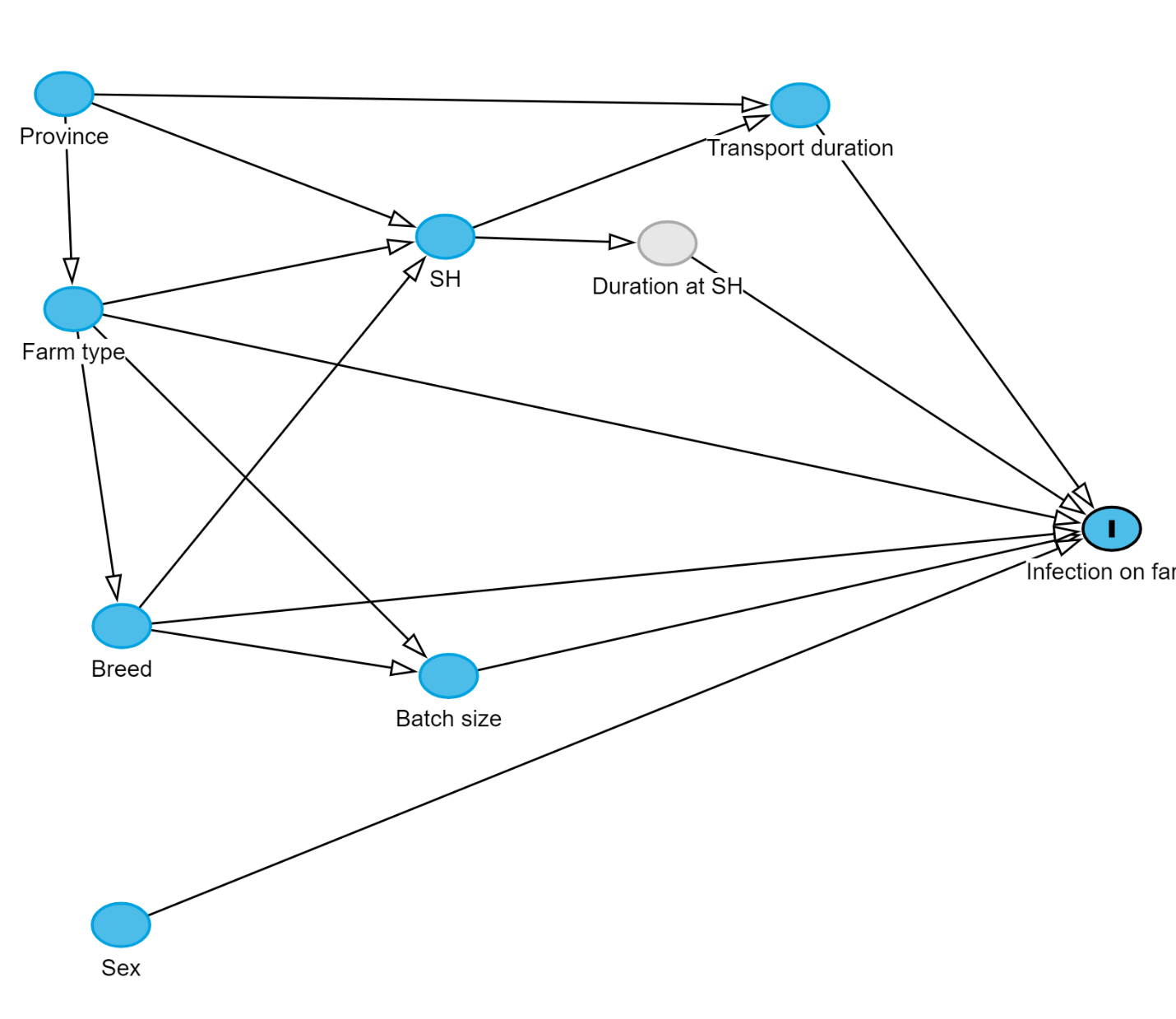
***

**Figure S1. Directed acyclic graph for the assumed causal relationship between explanatory variables and the outcome (infection on farm), which was approximated by the ELISA status.**


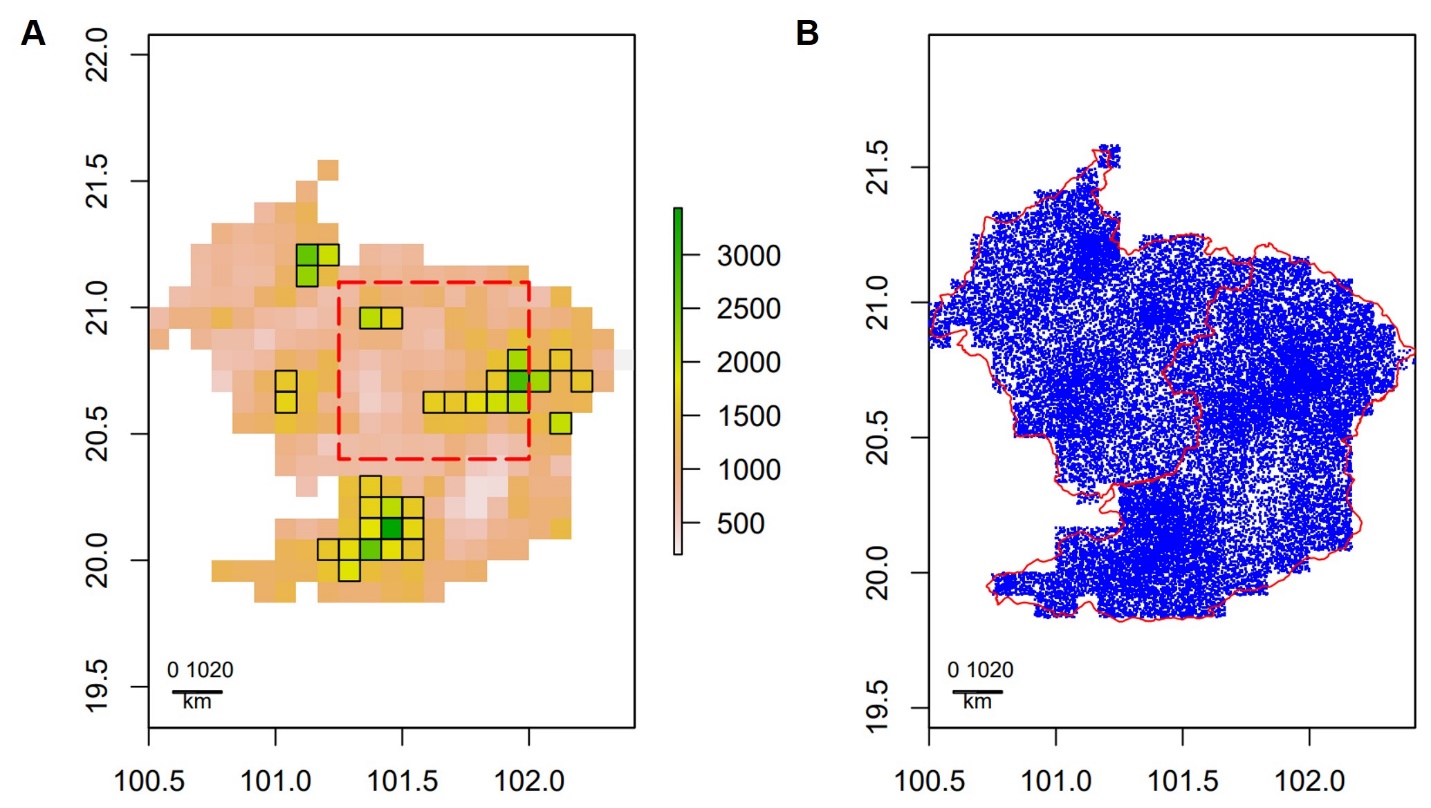


**
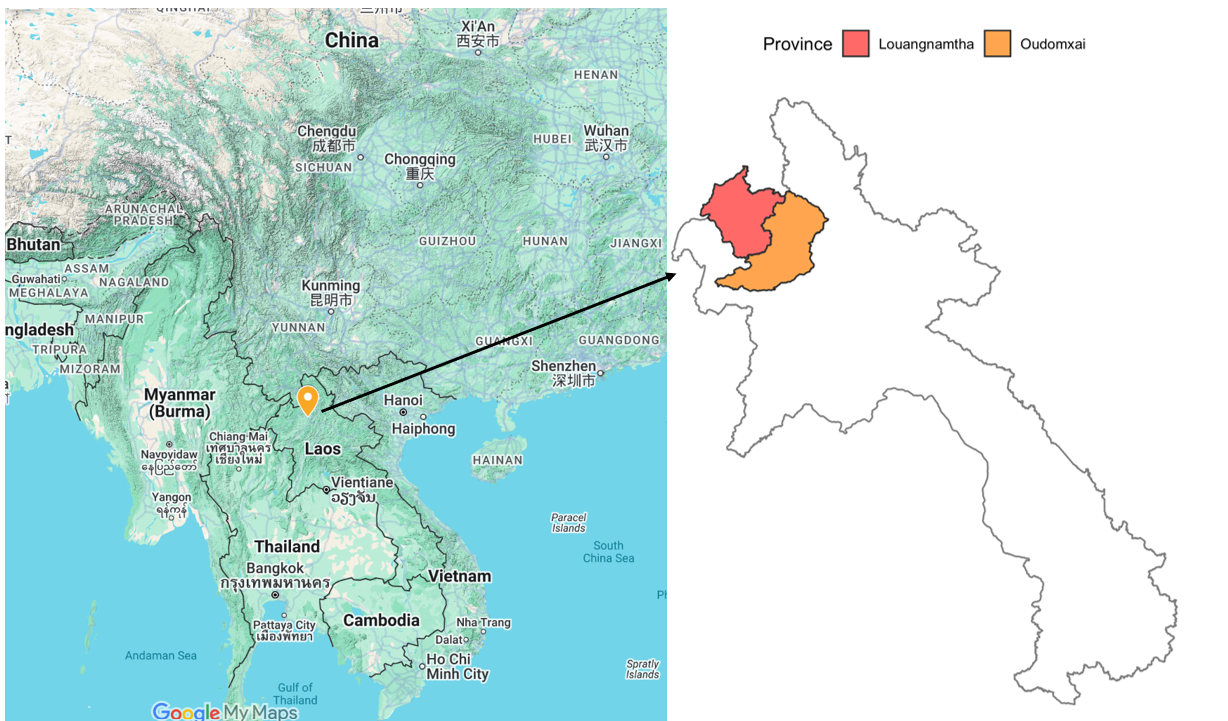
 C**

**Figure S2. Map of the study site.** (A) Pig densities within the study area based on Gridded Livestock of the World data. Grid cells defined as high pig density areas are marked with a black border and the selected simulation study area is shown as a red dashed box. (B) Distribution of simulated smallholder nodes. (C) Map of the study location (Oudomxay in orange and Luang Namtha in red) in relation to bordering countries


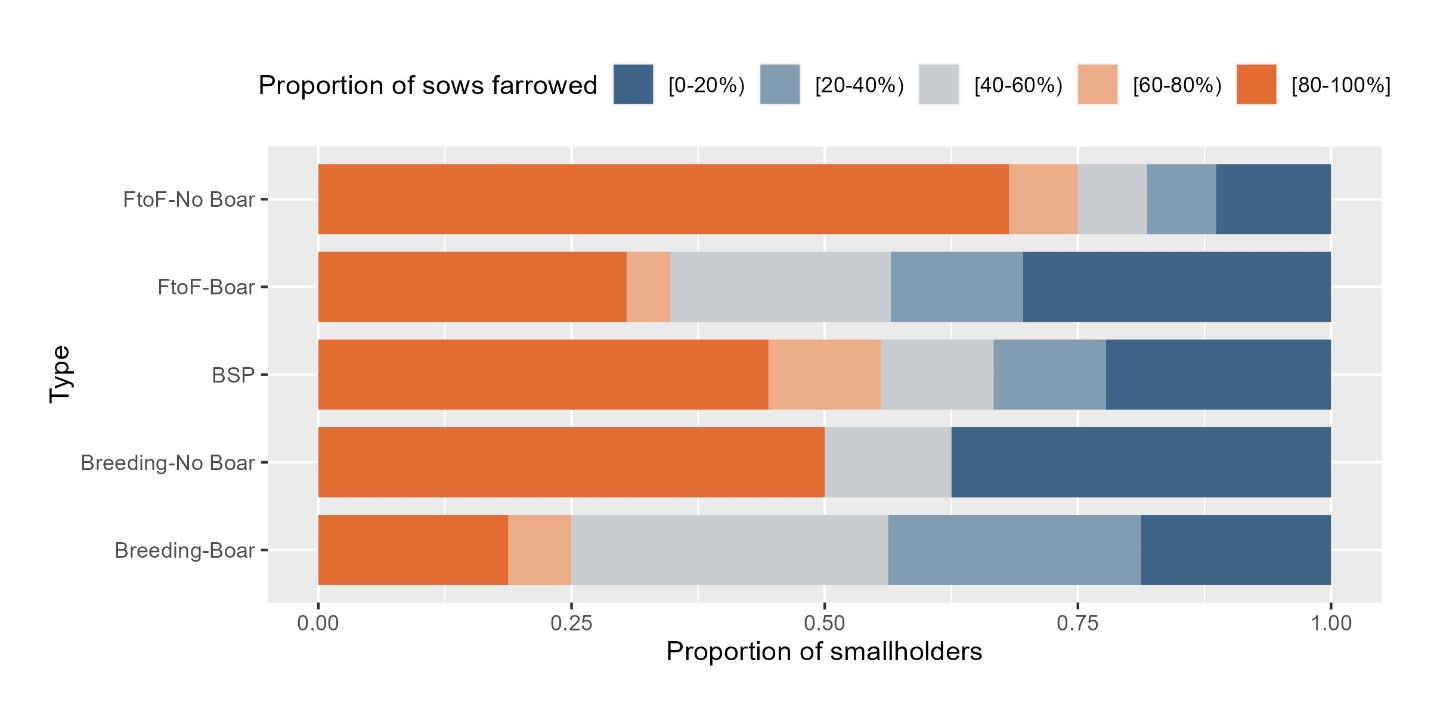


**Figure S3. Distributions of the proportion of sows that farrowed in the past 3 months stratified by smallholder type in northern Laos**. Each colour represents the category of the proportion of sows that farrowed in the past 3 months where [0-20%] indicates that less than 20% of sows in herd farrowed in the last 3 months etc.


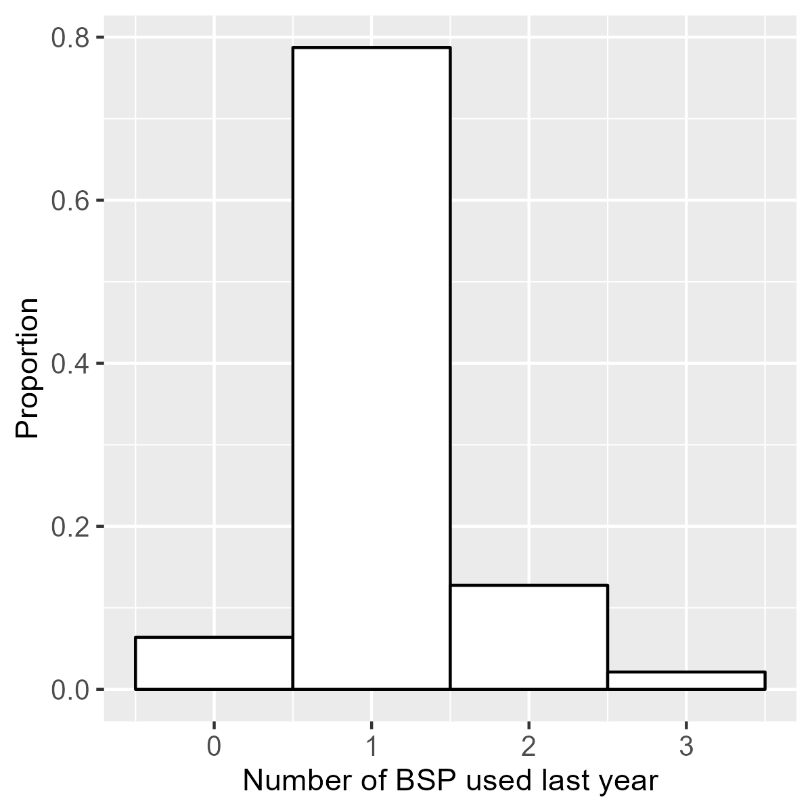


**Figure S4. Number of different boar service providers (BSPs) used by smallholders, who hired boars, in the past 1 year in northern Laos**

**
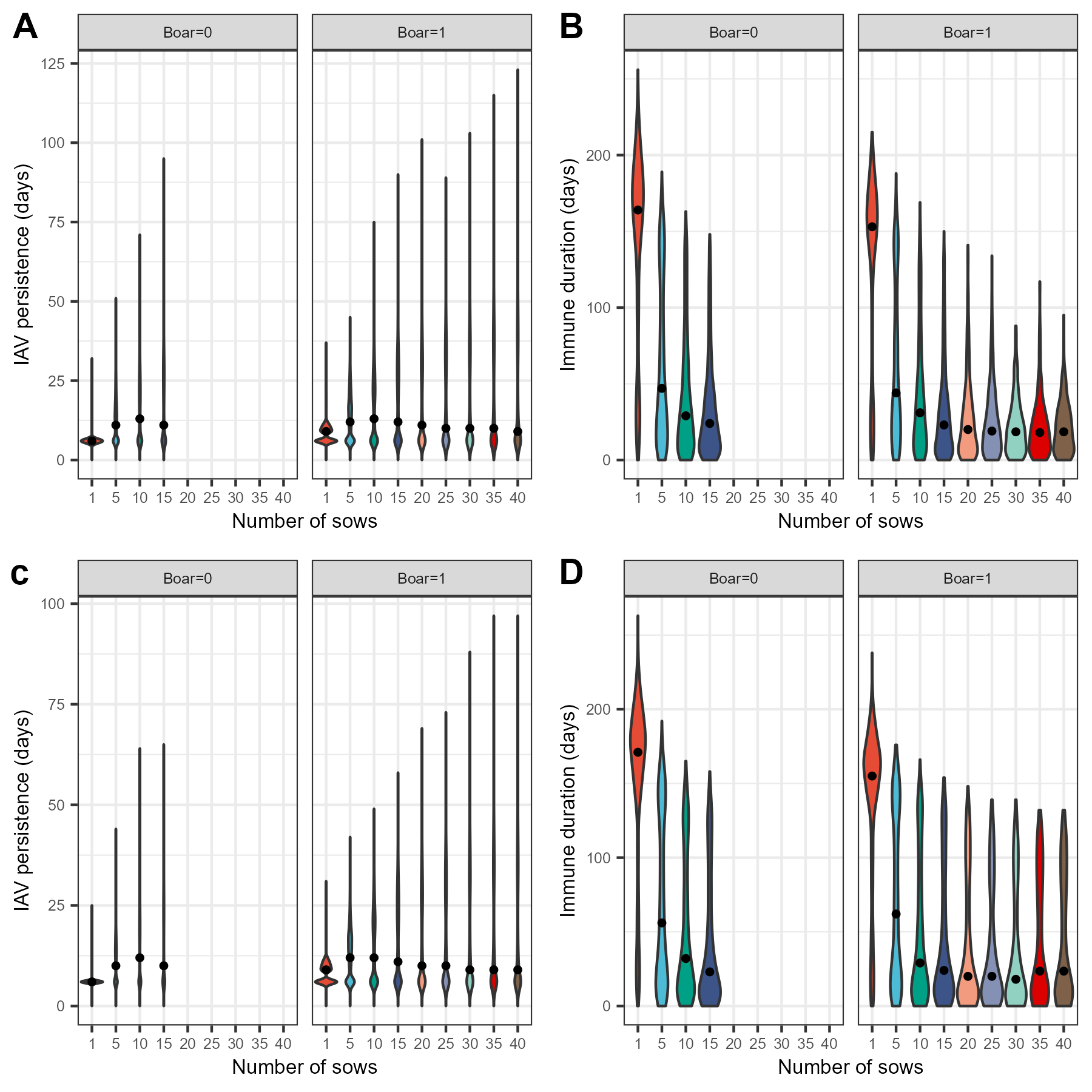
**

**Figure S5. Distributions of the simulated persistence and immune duration across smallholder type.** (A) IAV persistence and (B) immune duration of farrow-to-finish smallholders and (C) IAV persistence and (D) immune duration of breeding smallholders, stratified by the number of sows and boars


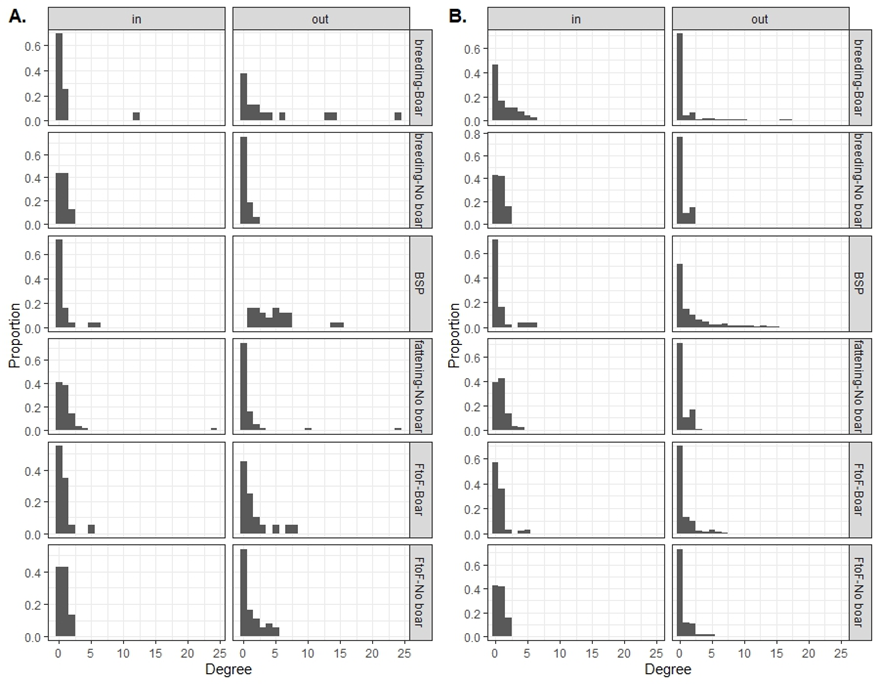


**Figure S6. Comparison of distributions of in- and out-degree by smallholder type. (A) observed pig trade data and (B) simulated pig trade network.**


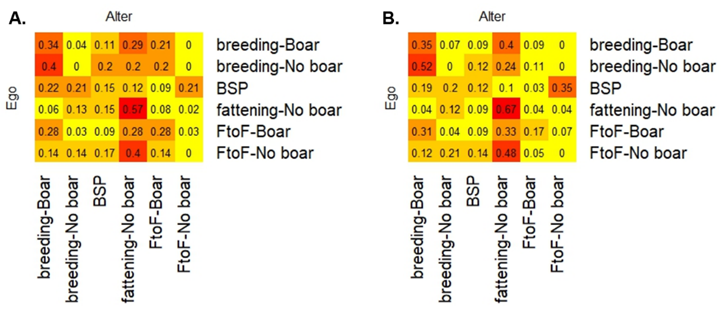


**Figure S7. Comparison of mixing matrices by smallholder type. (A) observed pig trade data and (B) simulated pig trade network. Cells represent, row-wise for each ego, the proportion of their contacts that belonged to each type.**


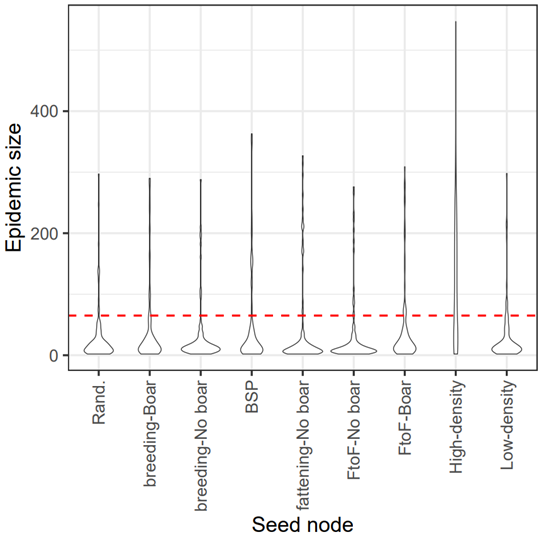


**Figure S8. Violin plot of the distribution of epidemic sizes following seeding in different smallholder types and pig density regions. (Rand = total random seeding). The selected cut-off of 65 infected nodes is shown (dotted red line)**


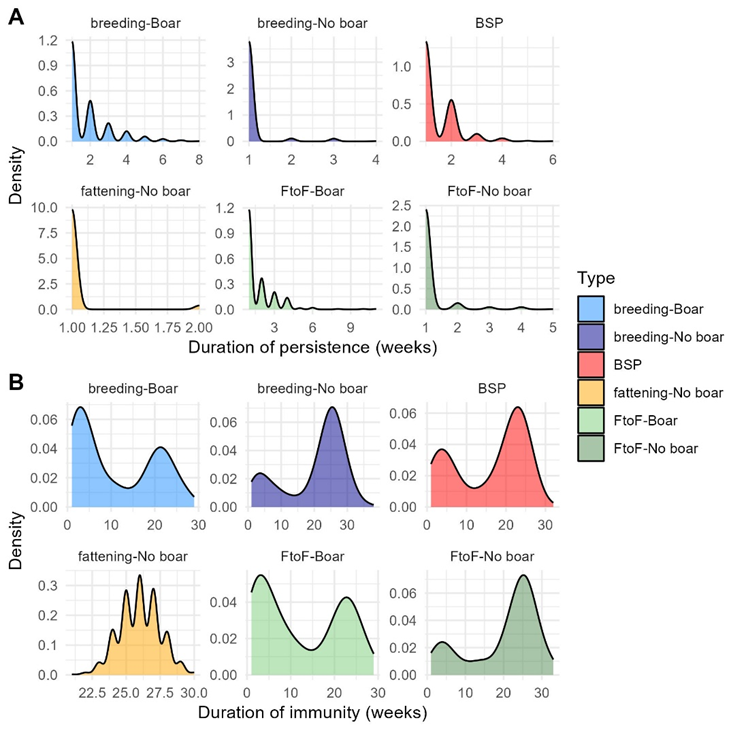


**Figure S9. Distributions of duration of node-level persistence (A) and immunity (B) of the simulated population of nodes.**


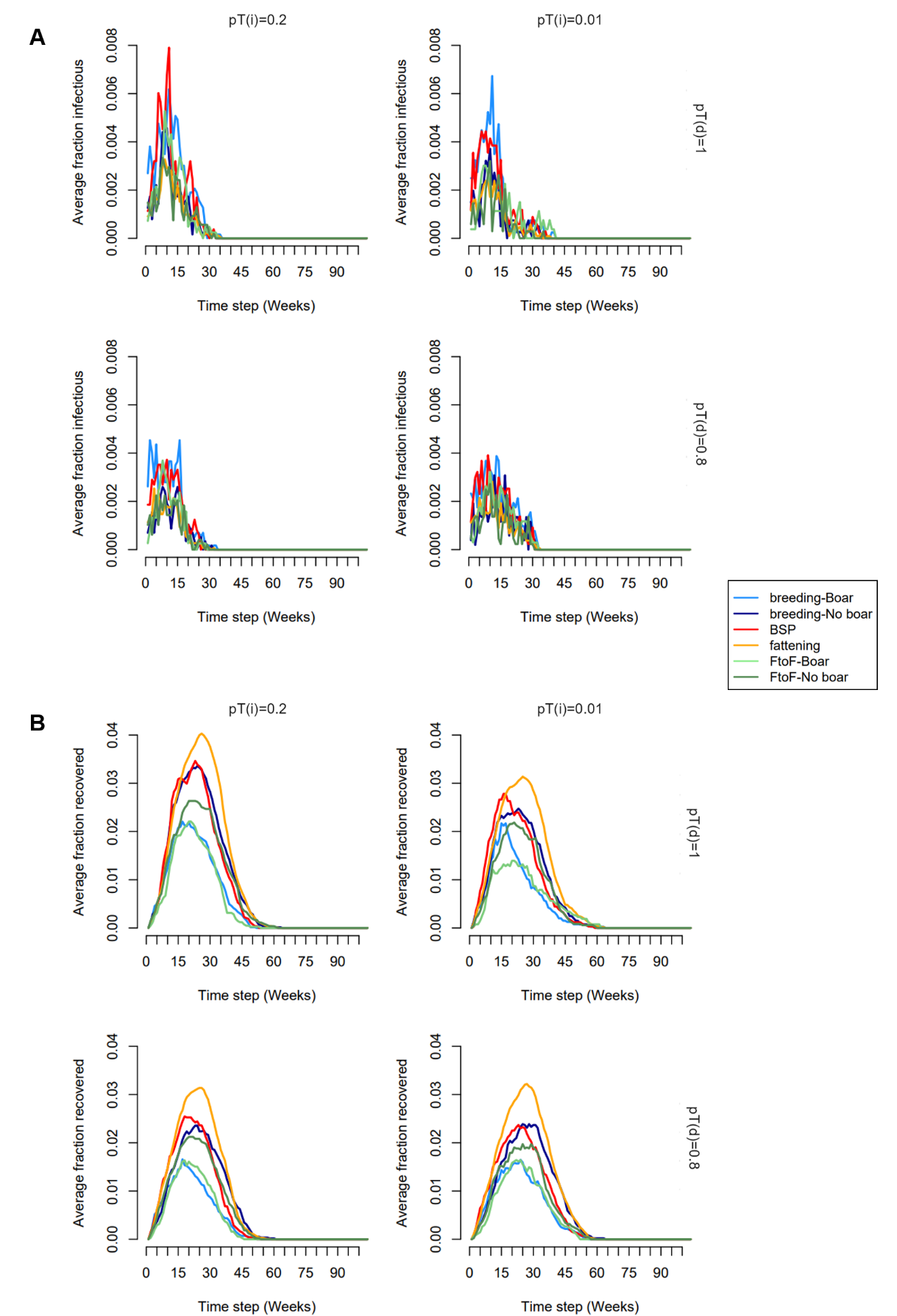


**Figure S10. Infectious disease dynamics by actor. The mean proportion of infectious (A) and recovered (B) nodes over the course of the simulation, shown here for all transmissibility scenarios following random actor seeding.**


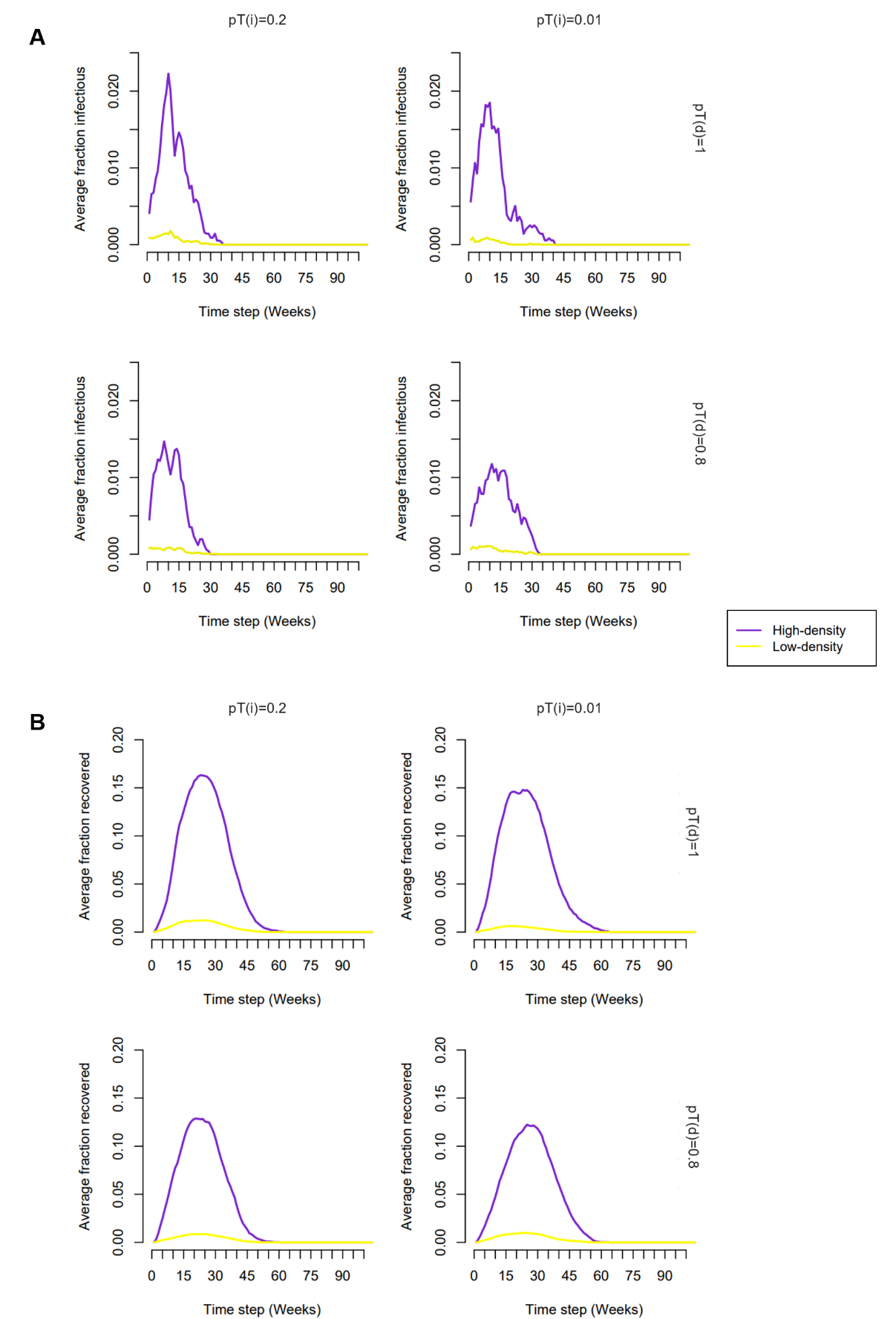


**Figure S11. Infectious disease dynamics by region. The mean proportion of infectious (A) and recovered (B) nodes over the course of the simulation, shown here for all transmissibility scenarios following random actor seeding.**


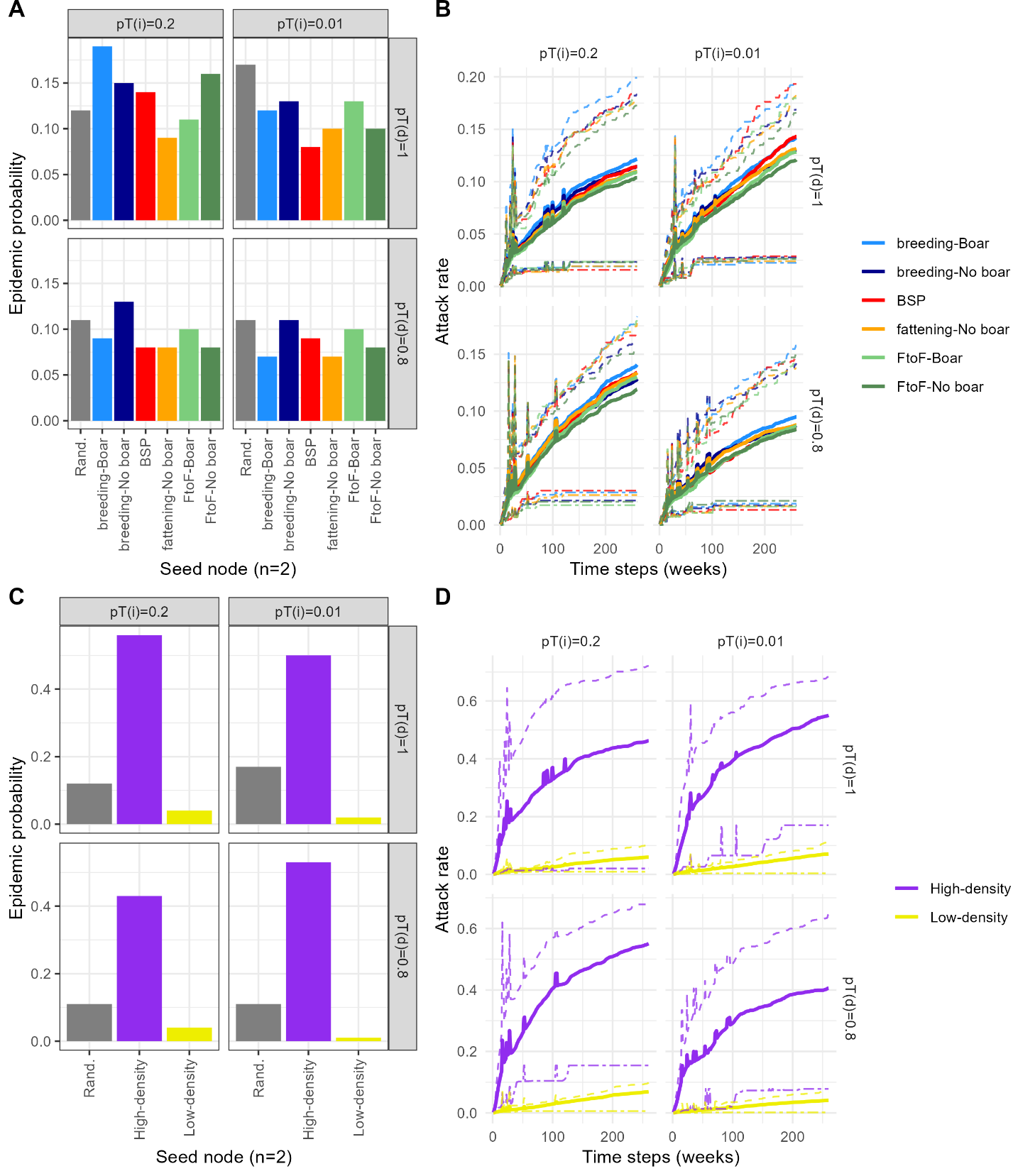
**Figure S12. Scenarios of persistently infected large commercial farms. Epidemic probability after seeding in defined smallholder types (A) and in different pig density regions (C) (Rand = total random seeding) and cumulative epidemic attack rate by smallholder type (B) and pig density region (D) following total random seeding. Solid lines show median cumulative epidemic attack rate; dashed lines show inter-95-percentiles across 100 iterations.**
